# Comparative Computational Analysis of Spike Protein Structural Stability in SARS-CoV-2 Omicron Subvariants

**DOI:** 10.3390/ijms242216069

**Published:** 2023-11-08

**Authors:** Anand Balupuri, Jeong-Min Kim, Kwang-Eun Choi, Jin Sun No, Il-Hwan Kim, Jee Eun Rhee, Eun-Jin Kim, Nam Sook Kang

**Affiliations:** 1 Graduate School of New Drug Discovery and Development, Chungnam National University, 99 Daehak-ro, Yuseong-gu, Daejeon 34134, Republic of Korea; abalupuri@cnu.ac.kr (A.B.); hwendiv@naver.com (K.-E.C.); 2 Division of Emerging Infectious Diseases, Bureau of Infectious Disease Diagnosis Control, Korea Disease, Control and Prevention Agency, 187 Osongsaengmyeong 2-ro, Osong-eup, Heungdeok-gu, Cheongju-si 28159, Republic of Korea; jmkim97@korea.kr (J.-M.K.); njs2564@korea.kr (J.S.N.); ilhwan98@korea.kr (I.-H.K.); jerhee001@korea.kr (J.E.R.);

**Keywords:** SARS-CoV-2, Omicron, MD simulation, MM/PBSA

## Abstract

The continuous emergence of new severe acute respiratory syndrome coronavirus 2 (SARS-CoV-2) variants with multiple spike (S) protein mutations pose serious threats to current coronavirus disease 2019 (COVID-19) therapies. A comprehensive understanding of the structural stability of SARS-CoV-2 variants is vital for the development of effective therapeutic strategies as it can offer valuable insights into their potential impact on viral infectivity. S protein mediates a virus’ attachment to host cells by binding to angiotensin-converting enzyme 2 (ACE2) through its receptor-binding domain (RBD), and mutations in this protein can affect its stability and binding affinity. We analyzed S protein structural stability in various Omicron subvariants computationally. Notably, the S protein sequences analyzed in this work were obtained directly from our own sample collection. We evaluated the binding free energy between S protein and ACE2 in several complex forms. Additionally, we measured distances between the RBD of each chain in S protein to analyze conformational changes. Unlike most of the prior studies, we analyzed full-length S protein–ACE2 complexes instead of only RBD–ACE2 complexes. Omicron subvariants including BA.1, BA.2, BA.2.12.1, BA.4/BA.5, BA.2.75, BA.2.75_K147E, BA.4.6 and BA.4.6_N658S showed enhanced stability compared to wild type, potentially due to distinct S protein mutations. Among them, BA.2.75 and BA.4.6_N658S exhibited the highest and lowest level of stability, respectively.

## 1. Introduction

The ongoing coronavirus disease 2019 (COVID-19) pandemic caused by the severe acute respiratory syndrome coronavirus 2 (SARS-CoV-2) continues to pose significant challenges to global public health. Since its emergence in late 2019, the virus has undergone genetic changes, leading to the emergence of several variants with distinct characteristics. Understanding the structural stability of key viral components, such as the spike (S) protein, is crucial for comprehending the potential impact of these variants on viral infectivity, transmissibility and the effectiveness of therapeutic interventions. Variants that are more stable than their predecessors can be more difficult to combat because structural stability may have a role in transmissibility tendencies [1,2,3,4]. Consequently, it is crucial for researchers to gain a comprehensive understanding of the variations in structural stability among these variants. Studies have shown that mutations in the S protein, which binds to angiotensin-converting enzyme 2 (ACE2) on host cells through its receptor-binding domain (RBD), can affect its binding free energy and thus can influence viral entry into cells and transmission [5,6,7,8,9,10,11,12,13,14,15,16,17,18]. The primary focus of this study is to analyze the structural stability of the S protein in recent SARS-CoV-2 Omicron subvariants using computational methods and to evaluate their potential impact on viral infectivity and pathogenesis. Specifically, the study aims to investigate the structural stability of the S protein and its binding affinity to the ACE2 receptor in the presence of mutations found in various Omicron subvariants. The hypothesis is that these mutations can alter the structural stability and binding affinity of the S protein, potentially leading to changes in viral infectivity and pathogenesis. The study further seeks to identify Omicron subvariants that exhibit high stability and those that possess comparatively lower stability. In this study, ‘stability’ refers to the structural stability of the S protein. Previous studies have not extensively investigated the structural stability of Omicron subvariants and the ramifications of mutations on their structural stability. Additionally, previous research has primarily focused on analyzing the RBD–ACE2 complex instead of the full-length S protein–ACE2 complex, which may not accurately represent the structural stability of the virus. Furthermore, prior studies predominantly concentrated on single mutations, while new variants often have multiple mutations, which may have synergistic or antagonistic effects on the virus’ structural stability. This study aims to address these gaps in the existing literature. The motivation for conducting this study is to provide valuable insights into the potential impact of these variants on viral infectivity and pathogenesis as this information can aid in the development of effective therapeutic strategies against SARS-CoV-2 subvariants. 

As of 22 October 2023, the worldwide tally of confirmed cases has surpassed 771 million, with over 6 million deaths reported (https://www.who.int/emergencies/diseases/novel-coronavirus-2019/situation-reports, accessed on 1 November 2023). As SARS-CoV-2 continues to evolve, complete eradication of the virus is unlikely. It is anticipated that COVID-19 will eventually resemble seasonal influenza, characterized by milder symptoms. Moreover, the acquisition of layered immune protection by a substantial portion of the population increases the probability of attaining herd immunity [19,20]. Recently, Rahmani and colleagues conducted docking studies and suggested that phytoconstituents used in their study may be used to treat COVID-19. This may pave the way for the future development of more effective natural antivirals against COVID-19 [21]. In the battle against COVID-19, the Omicron variant has proven to be a difficult opponent. Omicron emerged in late 2021 and spread rapidly around the world. Omicron is considered a variant of concern primarily due to its significant decrease in vaccine effectiveness, enhanced transmissibility, elevated risks of reinfection and immune evasion [22,23,24]. Several Omicron subvariants have subsequently emerged and these subvariants pose severe challenges to the efficacy of currently available vaccines and antibody therapeutics. The most concerning aspect of these new strains is that they appear to be more transmissible than previous SARS-CoV-2 strains [25,26,27,28]. Scientists around the world have been studying Omicron subvariants to better understand their characteristics and develop strategies for preventing future outbreaks [14,20,28,29,30,31,32,33,34,35]. Researchers have found that several mutations present within these new viruses may contribute towards increased transmission as well as enhanced resistance against current vaccines or treatments available for COVID19 infection [5,6,7,8,9,10,11,12,13,14,15,16,17,18]. Additionally, some individuals infected with one variant could become reinfected with another variant due to genetic differences between them. The pathogenesis of SARS-CoV-2 begins with the binding of the S protein to the ACE2. This binding event triggers a cascade of events that lead to the activation of Toll-like receptors (TLRs), particularly TLR4, causing the proliferation and production of proinflammatory cytokines. The excessive production of these cytokines leads to a cytokine storm, resulting in widespread inflammation [36,37,38]. The structural stability of the S protein is of particular interest as it influences the binding to the ACE2 and subsequent viral infectivity [1,2,3,4]. In our previous works [39,40], we examined several SARS-CoV-2 variants including single mutant variants (D614G, D614A, L455F, F456L and Q787H), double (D614G/E484K), triple (D614G/E484K/N440K), B.1.620, Delta, Alpha, Mu and Omicron. In continuation with our earlier works, we investigated recently emerged Omicron (BA.1) subvariants (BA.2, BA.2.12.1, BA.4/BA.5, BA.2.75 and BA.4.6) in this study. Instead of relying on publicly available sequence data, the S protein sequences analyzed in this study were directly obtained from our own sample collection, which offers several advantages. Sequencing own samples allows us to capture the genetic diversity of the virus within specific populations. This is especially important when studying local or unique variants. Publicly available data may not be up-to-date and it might not reflect the most recent mutations or variants. Sequencing your own samples provides real-time information, which is crucial for monitoring the virus’s evolution. By generating own sequences, you can ensure the quality and accuracy of the data. This is important for research and clinical decision making. Own samples might reveal unique insights into the virus’ behavior in particular populations or regions, contributing to a better understanding of its spread and impact. Generating own data provides the opportunity to cross-validate findings from publicly available data, enhancing the robustness and credibility of our research. The protein sequence information of Omicron subvariants derived from Korean patient samples was utilized to model full-length S protein–ACE2 complexes in one-, two-, and three-open-complex forms for each variant. We performed molecular dynamics (MD) simulations and molecular mechanics/Poisson–Boltzmann surface area (MM/PBSA) calculations on the full-length S protein–ACE2 complexes to analyze the binding free energies of the complexes. While the binding energy between the S protein and ACE2 receptor does not directly report the intrinsic stability of the isolated S protein, it indirectly indicates the stability of the S protein by assessing the strength of their interaction. A lower binding energy suggests a more stable interaction between the two molecules, signifying they are held together more tightly. This indirectly implies that the S protein, as a component of this complex, is likely to maintain its structural integrity and conformation, making it less prone to structural changes. Therefore, low binding energy indirectly indicates that the S protein is effectively maintaining its stability within the complex. In contrast, a weaker binding with higher binding energy may suggest that the S protein is less stable within the complex. Additionally, we analyzed the conformational changes in trimeric S protein chains based on the distances between the RBD of each chain. The significance of this study lies in its potential to enhance our understanding of the SARS-CoV-2 virus and its variants. By analyzing the full-length S protein–ACE2 complex and multiple mutations simultaneously, this study provides a more comprehensive understanding of the virus’s structural stability. Moreover, the findings of this study have implications for developing effective therapies against SARS-CoV-2 variants, which is crucial in controlling the ongoing pandemic.

## 2. Results

### 2.1. SARS-CoV-2 S Protein Genome/Protein Analysis in COVID-19 Patients

We identified several Omicron (BA.1) subvariants (BA.2, BA.2.12.1, BA.4/BA.5, BA.2.75, BA.2.75_K147E, BA.4.6 and BA.4.6_N658S) by whole-genome sequence analysis of the SARS-CoV-2 S protein isolated from COVID-19 patients in South Korea. Supplementary Figure S1 provides a summary of deletions and mutations observed in the S protein of Omicron subvariants. BA.1 is characterized by deletions at positions ∆V69, ∆H70, ∆V143, ∆Y144, ∆Y145 and ∆N211. BA.2, BA.2.12.1, BA.2.75 and BA.2.75_K147E display deletions at positions ∆L24, ∆P25 and ∆P26. BA.4/BA.5, BA.4.6 and BA.4.6_N658S exhibit deletions at positions ∆L24, ∆P25, ∆P26, ∆V69 and ∆H70. All subvariants exhibit a common set of mutations in their S protein sequence, including G142D, S373P, S375F, K417N, N440K, S477N, T478K, E484A, Q498R, N501Y, Y505H, D614G, H655Y, N679K, P681H, N764K, D796Y, Q954H and N969K. Apart from that, all subvariants except BA.1 have additional mutations such as T19I, A27S, V213G, S371F, T376A, D405N and R408S. BA.1 is characterized by additional mutations including A67V, T95I, L212I, G339D, S371L, G446S, Q493R, G496S, T547K, N856K and L981F. BA.2 shows additional mutations of G339D and Q493R. BA.2.12.1 exhibits additional mutations of G339D, L452Q, Q493R and S704L. BA.2.75 shows additional mutations of W152R, F157L, I210V, G257S, G339H, G446S and N460K. BA.4/BA.5 displays additional mutations of G339D, L452R and F486V. BA.4.6 exhibits additional mutations of G339D, R346T, L452R and F486V. The sequences obtained from our own sample collections were found to be similar to publicly available sequences for majority of the studied variants. However, we observed two distinct variants of BA.2.75 as well as BA.4.6. BA.2.75_K147E possesses an additional mutation of K147E compared to BA.2.75. Similarly, BA.4.6_N658S has an extra mutation of N658S in comparison to BA.4.6. This finding emphasizes the importance of relying on our own sample collections as it allowed us to detect unique variants that may have been overlooked when depending solely on publicly accessible data. These dissimilarities provide valuable insights into the virus’ genetic diversity.

### 2.2. Binding Free Energy Analysis of Full-Length S Protein–ACE2 Complex

**Figure 1:**
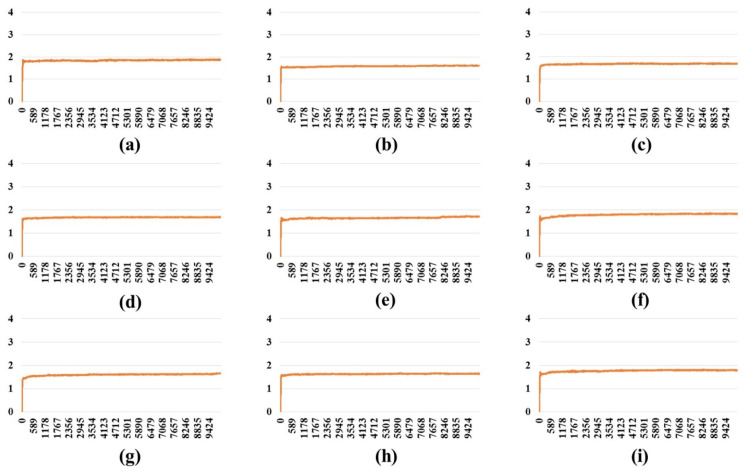
RMSD plot of the protein backbone atoms in the one-open-complex form during 10 ns MD simulation. (**a**) Wild type, (**b**) BA.1, (**c**) BA.2, (**d**) BA.2.12.1, (**e**) BA.4/BA.5, (**f**) BA.2.75, (**g**) BA.2.75_K147E, (**h**) BA.4.6 and (**i**) BA.4.6_N658S. *X* axis denotes the MD simulation time (ps) and *Y* axis denotes the RMSD value (nm).

**Figure 2:**
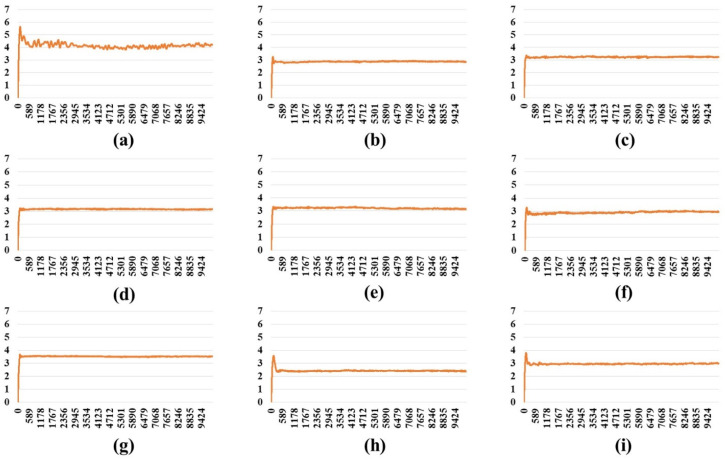
The RMSD plot of the protein backbone atoms in the two-open-complex form during 10 ns MD simulation. (**a**) Wild type, (**b**) BA.1, (**c**) BA.2, (**d**) BA.2.12.1, (**e**) BA.4/BA.5, (**f**) BA.2.75, (**g**) BA.2.75_K147E, (**h**) BA.4.6 and (**i**) BA.4.6_N658S. *X* axis denotes the MD simulation time (ps) and *Y* axis denotes the RMSD value (nm).

**Figure 3:**
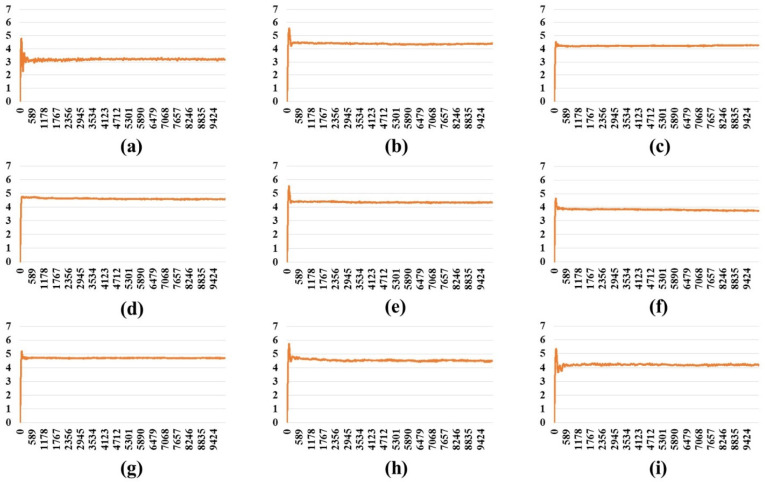
The RMSD plot of the protein backbone atoms in the three-open-complex form during 10 ns MD simulation. (**a**) Wild type, (**b**) BA.1, (**c**) BA.2, (**d**) BA.2.12.1, (**e**) BA.4/BA.5, (**f**) BA.2.75, (**g**) BA.2.75_K147E, (**h**) BA.4.6 and (**i**) BA.4.6_N658S. *X* axis denotes the MD simulation time (ps) and *Y* axis denotes the RMSD value (nm).

**Figure 4:**
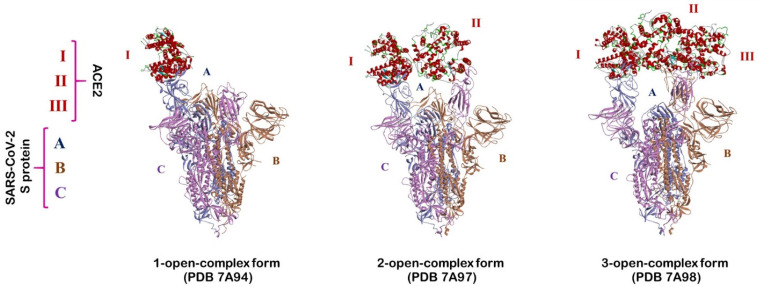
Structures of full-length SARS-CoV-2 S protein–ACE2 complex. The trimeric chains of the S protein are depicted in purple, brown and magenta colors, denoted as A, B and C, respectively. The ACE2 receptors, denoted as I, II and III, are depicted in the red color.

An MD simulation (10 ns) was carried out on the full-length S protein–ACE2 complex in a vacuum environment for each SARS-CoV-2 variant. The RMSD of the protein backbone atoms with respect to the starting structure was monitored during the simulations to ensure the stability of the simulated systems. RMSD plots (Figure 1, Figure 2 and Figure 3) demonstrate that simulated systems achieved convergence and were reasonably stable throughout the simulations in all (one-, two-, and three-open-complex) forms. The binding free energy between the full-length S protein and ACE2 was estimated with the MM/PBSA analysis. The total energy (E) included van der Waals (V) energy and electrostatic (E) energies. The binding free energy was computed between A–I chains for the one-open-complex form, A–I and B–II chains for two-open-complex form and A–I, B–II and C–III chains for three-open complex form (Figure 4, Supplementary Tables S1–S3). The average total binding free energy (ΔG) for each variant was determined through the summation of the total binding free energies associated with the one-, two-, and three-open-complex forms. Subsequently, the resulting sum was divided by three to obtain the average value. The plots for the binding free energy between various chains in the one-, two-, and three-open-complex forms are presented in Supplementary Figures S2–S4, respectively. The total binding free energy of the one-, two-, and three-open-complex forms and ΔG values are provided in Table 1. 

All Omicron subvariants were found to be more stable than the wild type in all open-complex forms. The ΔG values indicated that BA.2.75, BA.4/BA.5 and BA.1 were highly stable variants, while BA.2.12.1 and BA.2 were moderately stable. On the other hand, BA.2.75_K147E, BA.4.6 and BA.4.6_N658S were identified as less stable variants. Among Omicron subvariants, BA.2.75 and BA.4.6_N658S were found to be the most and the least stable variants, respectively. Specifically, BA.2.75 demonstrated a 2.4-fold increase in stability, whereas BA.4.6_N658S displayed a 1.7-fold increase in stability compared to the wild type. Interestingly, addition of the K147E mutation drastically reduced the BA.2.75 stability. Similarly, addition of the N658S mutation slightly reduced the BA.4.6 stability. Variations in stability among Omicron subvariants can influence viral entry, infection and antibody evasion. The high-to-moderate stability observed in BA.2.75, BA.4/BA.5, BA.1, BA.2.12.1 and BA.2 suggests that these subvariants may have an increased ability to attach to and enter host cells compared to BA.2.75_K147E, BA.4.6 and BA.4.6_N658S. This is supported by recent experimental studies which demonstrated that BA.2.75, BA.4/BA.5, BA.1, BA.2.12.1 and BA.2 possess high ACE2 binding affinities [11,12,13,14,15,16,17,41]. Furthermore, BA.2.75, BA.4/BA.5 and BA.2.12.1 display high transmissibility and the ability to evade antibodies [11,13,14,15,20,30].

The wild type demonstrated a ΔG value of −16,638.2 kJ/mol. The most stable variant, BA.2.75, exhibited a ΔG value of −39,834.9 kJ/mol while the least stable variant, BA.4.6_N658S, displayed ΔG value of −27,815.2 kJ/mol, respectively. Other variants including BA.4/BA.5, BA.1, BA.2.12.1, BA.2, BA.2.75_K147E and BA.4.6 exhibited ΔG values of −33,311.9, −33,180.5, −32,816.3, −32,773.1, −31,288.9 and −30,634.1 kJ/mol, respectively. The significantly higher ΔG value of BA.2.75_K147E (−31,288.9 kJ/mol) as compared to ΔG value of BA.2.75 (−39,834.9 kJ/mol) revealed that the addition of the K147E mutation significantly decreased the stability of BA.2.75. Similarly, the slightly higher ΔG value of BA.4.6_N658S (−27,815.2 kJ/mol) as compared to the ΔG value of BA.4.6 (−30,634.1 kJ/mol) showed that the addition of the N658S mutation slightly decreased the stability of BA.4.6. We calculated the contribution of residues to the binding free energy to understand the energy difference between BA.2.75 and BA.2.75_K147E, and between BA.4.6 and BA.4.6_N658S. Comparison of the BA.2.75 and BA.2.75_K147E variants revealed a huge difference in the energy contribution of residue 147 in all (one-, two-, and three-open-complex) forms (Supplementary Figures S5–S7). LYS-147 contributed favorably to the binding free energy of BA.2.75, while GLU-147 contributed unfavorably to the binding free energy of BA.2.75_K147E. Additionally, the K147E mutation also effected the energy contributions of nearby residues. This suggested that the K147E mutation significantly impacted the intramolecular interactions of the S protein. Salt–bridge interactions were not detected for LYS-147 in BA.2.75 in all open-complex forms; however, GLU-147 in BA.2.75_K147E showed salt–bridge interactions with LYS-150 and LYS-182 in all open-complex forms for various chains (Supplementary Figure S8). Furthermore, hydrogen bond interaction differences were observed for residue 147 in the three-open-complex form of BA.2.75 and BA.2.75_K147E. LYS-147 in BA.2.75 displayed hydrogen bond interactions with GLU-549 (occupancy = 95.2%) and ASN-572 (occupancy = 86.9%) in chain A, ALA-384 (occupancy = 74.0%) in chain B, and ASN-572 (occupancy = 73.2%) and GLU-571 (occupancy = 73.2%) in chain C. On the other hand, GLU-147 in BA.2.75_K147E showed hydrogen bond interactions with GLN-221 (occupancy = 82.3%) in chain A and ILE-88 (occupancy = 92.4%), GLU-87 (occupancy = 68.4%) and LEU-85 (occupancy = 54.5%) in chain B (Supplementary Figure S9). In the one- and two-open-complex forms, residue 147 showed no significant hydrogen bond interactions (occupancy > 50%). The K147E mutation in the BA.2.75_K147E variant caused intramolecular interaction differences that imply structural changes in the S protein. These changes may have impacted the binding free energy between the S protein and ACE2. A comparison of the contribution of residues to the binding free energy between the BA.4.6 and BA.4.6_N658S variants revealed an insignificant difference in the energy contribution of residue 658 in all (one-, two-, and three-open-complex) forms (Supplementary Figures S10–S12). Due to their polar uncharged side chains, asparagine and serine are unable to form salt bridges. Furthermore, no significant hydrogen bond interactions (occupancy > 50%) were observed for residue 658 in both the BA.4.6 and BA.4.6_N658S variants. The discrepancy in the binding free energy of BA.4.6 and BA.4.6_N658S variants may be attributed to the intramolecular interactions and energy contributions of residues other than residue 658, such as LYS-77, LYS-147, LYS-150 and LYS-182, as evident from Supplementary Figures S10–S12.

### 2.3. Stability Analysis of RBDs in Full-Length Trimeric S Protein Chains

**Figure 5:**
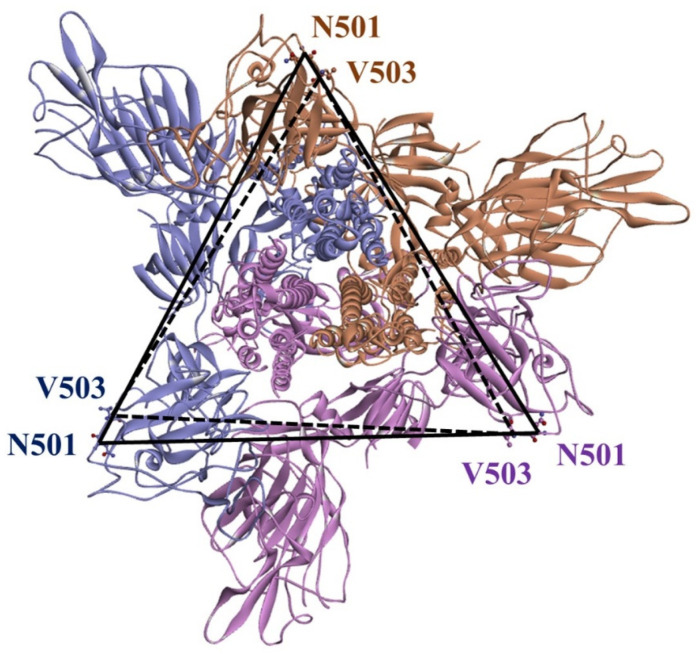
A representation of SARS-CoV-2 binding interface residues N501 and V503 forming the threefold symmetry with the same distance among trimeric RBD chains in the closed (PDB 6VXX) and 3-open-complex (PDB 7A98) forms. Trimeric chains (A, B and C) of S protein are shown in purple, brown and magenta colors, respectively.

**Figure 6:**
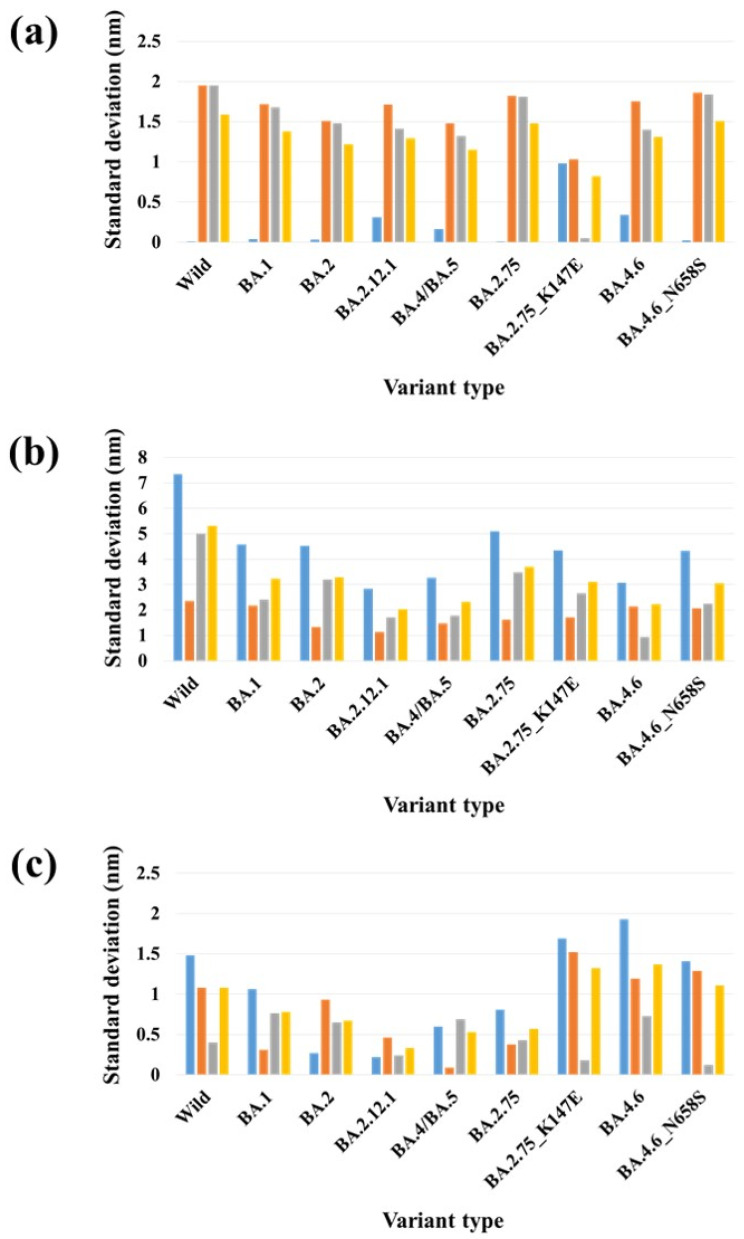
The standard deviations for distances between V503 residues in each chain for the final MD trajectory (10 ns). (**a**) One-open-complex form, (**b**) two-open-complex form and (**c**) three-open-complex form. *X* axis denotes the variant type and *Y* axis denotes the standard deviation for distance (nm). Blue indicates the standard deviation (SD) between the AB and BC chains, orange indicates the SD between the AB and AC chains, gray indicates the SD between the AC and BC chains and yellow indicates the SD among the A, B, and C chains.

**Figure 7:**
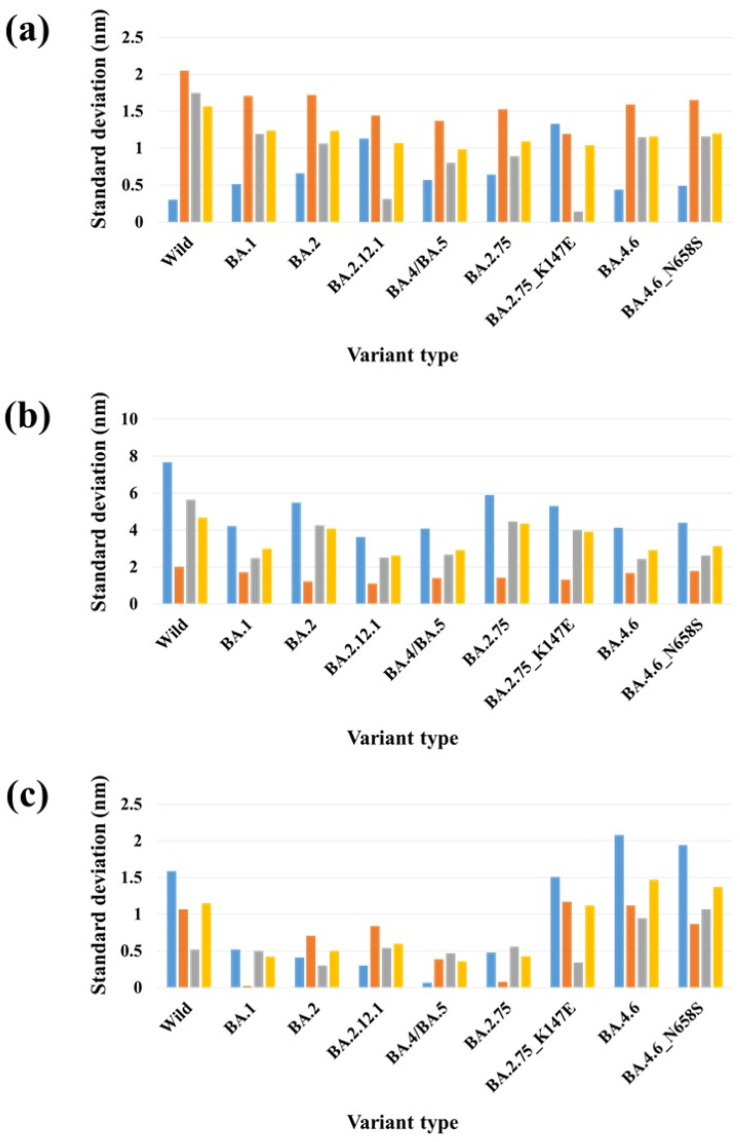
The standard deviations for distances between N501 residues in each chain of the S protein for the final MD trajectory (10 ns). (**a**) One-open-complex form, (**b**) two-open-complex form and (**c**) three-open-complex form. *X* axis denotes the variant type and *Y* axis denotes the standard deviation for distance (nm). Blue indicates the standard deviation (SD) between the AB and BC chains, orange indicates the SD between the AB and AC chains, gray indicates the SD between the AC and BC chains and yellow indicates the SD among the A, B, and C chains.

Mutations in the S protein of SARS-CoV-2, particularly in the ACE2 binding surface, can change the overall conformation of the S protein [42,43] and affect its binding with ACE2 and antibodies. Experimental studies have demonstrated that the Omicron subvariants exhibit different conformations of the RBD within the S protein, resulting in varying interactions with ACE2 [44]. However, the specific driving force behind this structural diversity remains unclear. It is speculated that the mutation patterns in the S protein of Omicron subvariants may contribute to this phenomenon. Further investigation is needed to gain a better understanding of the impact of these mutations on the structural diversity of the S protein [45]. Recently, Das et al. computationally analyzed the conformational stability of the complexes of monoclonal antibodies (mAbs) with the mutant S proteins of Omicron subvariants. They observed conformational changes in both mAb and the mutant S protein during the formation of the mAb–S protein complex. Collectively, their study indicated that the binding of therapeutic mAb to the mutant S protein induces a conformational change that further stabilizes the binding interface [46]. The SARS-CoV-2 S protein RBD residue V503 forms a symmetry among trimeric chains (A, B and C) with a distance below 1.0 nm in the closed (PDB 6VXX) [47]. Similarly, another RBD residue, N501, forms symmetry among trimeric chains with distance of 8.7 nm in the three-open-complex form (PDB 7A98) [48] (Figure 5). Similar to our previous studies [39,40], we analyzed the stability of trimeric S protein chains based on the distances and standard deviation (SD) for the distances between V503 residues and between N501 residues among trimeric chains. Table 2 and Figure 6 summarize the distance and SD values for V503 in one-, two-, and three-open-complex forms in the final MD trajectory. Supplementary Figures S13–S15 display the SD values for V503 residues in all MD trajectories. Table 3 and Figure 7 summarize the distance and SD values for N501 in one-, two-, and three-open-complex forms in the final MD trajectory. Supplementary Figures S16–S18 display the SD values for N501 residues in all MD trajectories. 

A similar pattern was observed for distance and SD values between V503 residues and between N501 residues. In the one- and two-open-complex forms, all Omicron subvariants showed commonly lower distance and SD values between V503 residues and between N501 residues compared to the wild type. However, in the three-open-complex form, the distance and SD values between V503 residues and between N501 residues compared to the wild type were higher for less stable variants (BA.4.6 and BA.4.6_N658S) than for the highly stable (BA.2.75, BA.4/BA.5 and BA.1) and moderately stable (BA.2.12.1 and BA.2) variants. Another less stable variant, BA.2.75_K147E, displayed higher distance and SD values between V503 residues compared to the wild type, while exhibiting comparable distance and SD values between N501 residues. Our results suggest that variants with lower stability exhibit a greater deviation in their RBDs compared to variants with higher stability. The observed differences in the RBD deviations between variants with lower and higher stability imply structural variations that potentially contribute to differences in their intramolecular interactions and binding free energies with ACE2.

In the one-open-complex form, the SD value for V503 residues in the wild type was 1.59 nm, while SD values for the variants ranged from 0.82 to 1.51. In the two-open-complex form, the SD value for V503 residues in the wild type was 5.31 nm, whereas SD values for the variants ranged from 2.02 to 3.69. In the three-open-complex form, the SD value for V503 residues in the wild type was 1.08 nm. SD values for V503 residues in BA.2.75, BA.4/BA.5, BA.1, BA.2.12.1 and BA.2 ranged from 0.33 to 0.78; however, SD values for BA.2.75_K147E, BA.4.6 and BA.4.6_N658S ranged from 1.11 to 1.37. SD values for N501 residues in the wild type in one- and two-open-complex forms were 1.57 and 4.66 nm, respectively. SD values for N501 residues in variants ranged from 0.98 to 1.24 in the one-open-complex form, whereas SD values for variants ranged from 2.62 to 4.35 in the two-open-complex form. In the three-open-complex form, the SD value for N501 residues in the wild type was 1.15 nm, while SD values for BA.4.6, BA.4.6_N658S and BA.2.75_K147E were 1.47, 1.37 and 1.12 nm, respectively. 

A comparison of the distance and SD values between BA.2.75 and BA.2.75_K147E suggested structural changes between these variants and supports the interaction and ΔG differences. SD values for V503 residues of BA.2.75 in the one-, two-, and three-open-complex forms were 1.48, 3.69 and 0.57 nm, respectively; however, SD values for V503 residues of BA.2.75_K147E in one-, two-, and three-open-complex forms were 0.82, 3.10 and 1.32 nm, respectively (Table 2). Similarly, SD values for N501 residues of BA.2.75 in the one-, two- and three-open-complex forms were 1.09, 4.35 and 0.43 nm, respectively; however, SD values for N501 residues of BA.2.75_K147E in the one-, two- and three-open-complex forms were 1.04, 3.92 and 1.12 nm, respectively (Table 3). A comparison between BA.4.6 and BA.4.6_N658S showed small differences in the distance and SD values for both V503 and N501 residues in all (one-, two-, and three-open-complex) forms. This is in agreement with the interaction and ΔG differences between these variants. SD values for V503 residues of BA.4.6 in one-, two-, and three-open-complex forms were 1.31, 2.23 and 1.37 nm, respectively; however, SD values for V503 residues of BA.4.6_N658S in one-, two- and three-open-complex forms were 1.51, 3.06 and 1.11 nm, respectively (Table 2). Similarly, SD values for N501 residues of BA.4.6 in one-, two- and three-open-complex forms were 1.16, 2.93 and 1.47 nm, respectively; however, SD values for N501 of BA.4.6_N658S in one-, two- and three-open-complex forms were 1.20, 3.13 and 1.37 nm, respectively (Table 3).

## 3. Discussion

The recent emergence of Omicron subvariants, characterized by mutations in the S protein, particularly within the RBD, poses a significant challenge to existing treatment strategies. These variants not only exhibit resistance to neutralization by antibodies generated through vaccination or prior infection with SARS-CoV-2 but also demonstrate remarkably high transmissibility. This combination of reduced antibody efficacy and increased transmissibility highlights the need for further research and development of alternative approaches to effectively combat the Omicron subvariants. Recently, various human therapeutic mAbs such as adintrevimab, beludavimab and regdanivimab were suggested as potential candidates for treating Omicron subvariants using in silico approaches [46]. 

The Korea Disease Control and Prevention Agency (KDCA) is closely tracking and investigating SARS-CoV-2 variants in Korean patients. Previously, the KDCA identified several SARS-CoV-2 variants including single mutant variants (D614G, D614A, L455F, F456L and Q787H), double (D614G/E484K), triple (D614G/E484K/N440K), B.1.620, Delta, Alpha, Mu and Omicron. We investigated these variants in our previous works [39,40]. Recently, the KDCA identified various Omicron subvariants including BA.1, BA.2, BA.2.12.1, BA.4/BA.5, BA.2.75, BA.2.75_K147E, BA.4.6 and BA.4.6_N658S. These variants exhibit multiple mutations both within and outside the RBD of the S protein. Additionally, these variants are also characterized by specific deletions in their S protein sequences (Supplementary Figure S1). We investigated newly identified Omicron subvariants in this study. Protein sequence information of Omicron subvariants was utilized to model full-length S protein–ACE2 complexes in one-, two-, and three-open-complex forms for each variant. SARS-CoV-2 can exist in the gas phase/as aerosols and can remain stable (viable and infectious) on different surfaces such as plastic, stainless steel, copper and cardboard for several days [49]. Furthermore, SARS-CoV-2 infects hosts from the outer air to inner respiratory systems. Accordingly, we performed MD simulations on the full-length S protein–ACE2 complex of various Omicron subvariants in the gas phase, similar to our previous studies [39,40]. Furthermore, MM/PBSA calculations were carried out to analyze the binding free energy of the complexes. Due to the large size of the simulated system and limited computing facilities, we simulated each complex for 10 ns. This simulation time is short as compared to current MD standards. Thus, these simulations may be susceptible to insufficient sampling of protein conformations. Furthermore, simulating proteins in vacuum neglects the effects of solvation, which can affect the protein’s conformation and stability. However, short MD simulations in vacuum can be useful for comparative analysis of different proteins or variants, as long as the same conditions and parameters are used for each simulation. This can provide insights into the differences between proteins, without the added complexity of solvent effects. 

Beyond the discussed limitations of this study, binding free energy values suggested Omicron subvariants to be more stable than the wild type. This aligns with the findings of an experimental investigation through protein binding assay using Microscale thermophoresis (MST), which revealed that the Omicron variant exhibits a five-fold greater affinity compared to the wild type [18]. Furthermore, this is in agreement with other computational studies which suggested that the Omicron variant binds to ACE2 more strongly than the wild type [50,51,52]. BA.2.75 and BA.4.6_N658S were found to be the most and the least stable variants, respectively. Additionally, BA.4/BA.5, BA.1, BA.2.12.1 and BA.2 showed high-to-moderate stability, while BA.4.6 and BA.2.75_K147E displayed low stability. This observation is consistent with the findings of recent experimental studies where the binding affinity between ACE2 and the S protein of Omicron subvariants was assessed using surface plasmon resonance (SPR). BA.2.75 showed the strongest ACE2 binding among the studied subvariants [11,12]. Also, BA.4/BA.5, BA.1, BA.2.12.1 and BA.2 demonstrated high ACE2 binding affinities [12,13,14,15,16,17]. These findings are in line with previous experimental research that indicated that the BA.2.75, BA.1 and BA.2 sub-lineages of Omicron demonstrate enhanced viral entry capabilities [41]. Furthermore, our results are in agreement with several recent experimental studies which reported that BA.4/BA.5 and BA.2.12.1 exhibit high transmissibility and escape vaccine- and infection-induced antibodies [13,14,15,20,30]. Cao et al. investigated the transmissibility and antibody evasion capabilities of different SARS-CoV-2 variants. The experimental results revealed that the variants BA.2.75, BA.2.12.1, BA.4 and BA.5 have high transmissibility. Additionally, these variants were found to evade antibodies [11,13]. Hachmann et al. evaluated the levels of neutralizing antibodies against different variants of SARS-CoV-2. The results demonstrated that BA.2.12.1, BA.4 and BA.5 subvariants possess a significant ability to evade neutralizing antibodies that are generated either through vaccination or prior infection [30]. Tuekprakhon et al. investigated the neutralization of the BA.4/5 variant using vaccine and naturally immune serum, as well as monoclonal antibodies. They found that BA.4/5 reduced neutralization by serum from vaccinated individuals. Similarly, serum from vaccine breakthrough infections also showed significant reductions in neutralization against BA.4/5, indicating a potential risk of repeat Omicron infections [15]. Wang et al. comprehensively evaluated the antigenic properties of BA.2.12.1 and BA.4/5 variants. BA.2.12.1 showed a modest level of resistance to sera from vaccinated and boosted individuals, whereas BA.4/5 exhibited significant resistance to the same sera [14].

Interestingly, addition of a single point mutation (K147E) in BA.2.75_K147E drastically reduced the stability, as compared to the most stable variant BA.2.75. LYS-147 contributed positively to the binding free energy of BA.2.75, whereas GLU-147 contributed unfavorably to the binding free energy of BA.2.75_K147E. The interaction analysis revealed that changes in the intramolecular interactions due to K147E mutation might be responsible for the decreased stability. On the other hand, addition of the N658S mutation slightly decreased the stability of BA.4.6_N658S compared to the less stable variant BA.4.6. There were no significant differences in the interaction observed for residue 658 between the BA.4.6 and BA.4.6_N658S variants. The disparity in binding free energy between the BA.4.6 and BA.4.6_N658S variants may be attributed to the intramolecular interactions and energy contributions of residues other than residue 658. Overall, these findings emphasize the significance of specific residues in contributing to the stability of the S protein–ACE2 interaction. Mutations at critical positions can have varying effects on the stability, depending on the specific residue and the nature of the mutation. Understanding these interactions and their effects on stability provides valuable insights for developing strategies to disrupt the S protein–ACE2 interaction. 

The structural stability of the SARS-CoV-2 S protein is crucial for its strong interaction with ACE2. Mutations in the S protein are anticipated to have a significant impact on the structural stability required for the interaction between SARS-CoV-2 and ACE2. Accordingly, we analyzed the conformational changes by calculating the distance and SD of distances between N501 residues and between V503 residues among trimeric chains of the S protein. V503 and N501 residues are located within the RBD region of the S protein and are part of the binding interface between the S protein and ACE2. These residues form a symmetry among trimeric chains in the closed (PDB 6VXX) [47] and open (PDB 7A98) [48] of the S protein. N501 plays a critical role in the binding interaction between the S protein and ACE2. It forms a hydrogen bond with ACE2, which stabilizes the binding and is essential for the virus to enter human cells [53]. The variations in the distance and SD of distances between V503 residues and between N501 residues suggest structural changes in the S protein, potentially affecting its binding with ACE2. The smaller distance and SD values observed in Omicron subvariants in both the one- and two-open-complex forms suggest reduced RBD deviation and enhanced stability of trimeric S protein chains compared to the wild type. This indicates small structural changes and a more tightly packed, rigid structure in the Omicron subvariants. The enhanced stability of the trimeric S protein chains in the Omicron subvariants can have significant implications for the overall structure and function of the complex. It may contribute to increased resistance against conformational changes or disruptions in the viral S protein. This could potentially make it more difficult for the immune system or therapeutic agents to target and neutralize the virus. Moreover, the enhanced stability of the trimeric S protein chains could impact the binding affinity and interactions with host cell receptors. The tighter packing and rigidity may lead to alterations in the binding interface, potentially affecting the ability of the virus to enter host cells or evade immune recognition. Overall, the smaller distance and SD values observed in the Omicron subvariants indicate a potentially more stable trimeric S protein structure. The less stable variants including BA.4.6_N658S, BA.4.6 and BA.2.75_K147E displayed higher distance and SD values, whereas more stable variants including BA.2.75 and BA.4/BA.5 showed lower distance and SD values as compared to the wild type in the three-open-complex form. The data indicate that the deviation of RBDs was greater in variants with lower stability compared to those with higher stability. This corresponds with the findings of crystallographic and cryo-electron microscopy (cryo-EM) structures, which revealed that BA.2.75 and BA.4/BA.5 possess a compact architecture characterized by a tight inter-subunit organization [12,13]. The significant disparities in distance and SD values between V503 residues and between N501 residues indicated notable structural variations between BA.2.75 and BA.2.75_K147E, substantiating dissimilarities in their intramolecular interactions and binding free energies with ACE2. The slight differences in the distance and SD values between V503 residues and between N501 residues suggested minor structural changes between BA.4.6 and BA.4.6_N658S, which support subtle differences in their intramolecular interactions and binding free energies with ACE2.

Overall, a comprehensive understanding of the structural stability of SARS-CoV-2 variants can offer valuable insights into their potential impact on viral infectivity, influencing disease progression and transmission rates. Variations in stability among SARS-CoV-2 variants may have implications for viral entry, infection and antibody evasion. More stable subvariants can efficiently attach to and enter host cells, while less stable subvariants may have reduced viral entry efficiency. Highly stable subvariants may evade immune responses by being less prone to antibody recognition and neutralization. The identification of more stable subvariants and less stable variants may guide the design of more effective drugs or vaccines that specifically target these variants, potentially leading to more effective treatments and preventive measures. This knowledge can empower scientists to prioritize vaccine candidates, ensuring a focused response to the most concerning variants. Variants with significant structural differences may necessitate the development of specific vaccine candidates or adaptations to existing vaccines.

## 4. Materials and Methods

### 4.1. Ethical Considerations

All procedures performed in studies involving human participants were in accordance with the ethical standards of the institutional and/or national research committee and with the 1964 Declaration of Helsinki and its later amendments or comparable ethical standards. This study was approved by the Institutional Review Board at the Korea Disease Control and Prevention Agency (2020-03-01-P-A) and is considered to be a public health act to the outbreak. Thus, the board has waived the requirement for written consent as outlined in the Title Laboratory Respondence to COVID-19. All the methods presented in this study were conducted in accordance with the relevant guidelines and regulations.

### 4.2. Genome/Protein Sequence Investigation of Korean Patients with COVID-19

Samples were selected for sequencing to maximize epidemiologic breadth. As such, samples were chosen based on the epidemiological links inferred from outbreak investigation data. We selected samples from sporadic cases, and we randomly selected a few representative samples from epidemiologically linked large outbreaks. Nasopharyngeal and oropharyngeal swab specimens were collected from symptomatic patients to detect SARS-CoV-2 by real time reverse transcriptase–polymerase chain reaction (RT-PCR). RNA was extracted from 140 μL of the specimens using a Qiagen viral RNA mini kit (Qiagen, Hilden, Germany) according to the manufacturer’s protocol. Next, real-time RT-PCR was performed on the cycle threshold value of the SARS-CoV-2 target gene (ORF 1b and E) [54]. For whole-genome sequencing, the cDNA was amplified using the QIAseq SARS-CoV-2 Primer Panel V3 and QIAseq FX DNA Library UDI Kit (QIAGEN, Hilden, Germany), and sequencing was performed on a MiSeq instrument using a MiSeq reagent kit V2 (Illumina, San Diego, CA, USA) to obtain an average genome coverage of >1000× for all samples. The reads were trimmed and mapped to the reference genome Wuhan-Hu-1 (GenBank: MN908947.3) using CLC Genomics Workbench version 20.0.3 (CLC Bio, Aarhus, Denmark). The lineages and clades of the SARS-CoV-2 sequences were assigned using Nextclade v1.7.1 [55] and PANGOLIN [56]. Genome/protein sequences whose QC was confirmed with a score of 0 to 29 (color green), indicating good quality in Nextclade, were used for analysis. Also, Missing data (N), Mixed sites (M), Stop codons (S) and Frame shifts (F), which are used as QC parameters, were checked.

### 4.3. SARS-CoV-2 S Protein Structure

The protein data bank (PDB) entries 7A94, 7A97 and 7A98 [48] correspond to different structures of the full-length SARS-CoV-2 S protein complexed with ACE2. Specifically, 7A94 represents the one-open-complex form with a single ACE2 molecule bound, 7A97 represents the two-open-complex form with two ACE2 molecules bound and 7A98 represents the three-open-complex form with three ACE2 molecules bound (Figure 4). Previously, we explored the structural stability of the S protein in other SARS-CoV-2 variants using models based on these specific structures. Similar to our previous works [39,40], we selected PDBs 7A94, 7A97 and 7A98 in this study. The structures were retrieved from the PDB and utilized as wild-type templates to model one-, two- and three-open-complex forms of BA.1, BA.2, BA.2.12.1, BA.4/BA.5, BA.2.75, BA.2.75_K147E, BA.4.6 and BA.4.6_N658S. SWISS-MODEL web server [57] was employed for the modeling of deletion mutants. Homology modeling in the SWISS-MODEL web server follows a well-established procedure to predict the three-dimensional structure of a protein based on homologous structures. We used ‘User Template’ mode for modeling. This mode only requires the amino acid sequence of the target protein and template structure as inputs. A full-length SARS-CoV-2 S protein sequence with specific deletions was used as the input, while each open-complex PDB was used as the template structure for homology modeling (Supplementary Figure S1). The automatic pipeline performs sequence alignment between the target protein and the template using BLAST [58] and HHblits algorithms [59]. The top-ranked alignment is automatically selected to build the model. SWISS-MODEL provides two key metrics, namely global model quality estimate (GMQE) [57] and QMEANDisCo global [60] scores, which are essential for assessing the quality of the generated models. These scores were utilized in our study to evaluate and determine the overall quality of the protein structure models. GMQE and QMEANDisCo global scores provide an overall assessment of model quality, ranging from 0 to 1. Higher scores indicate higher expected quality. GMQE and QMEANDisCo global scores above 0.7 indicate a high-quality model with acceptable accuracy and reliability. GMQE and QMEANDisCo global scores above 0.7 demonstrated both the accuracy and reliability of all the developed models. The point mutations were introduced using the ‘Build and Edit Protein’ module in Discovery Studio V22 (BIOVIA, San Diego, CA, USA).

### 4.4. MD Simulation 

MD simulations were performed similar to our previous works on SARS-CoV-2 variants [39,40]. Identical MD simulation conditions and parameters allow us to compare the Omicron variants reported in this study, as well as to compare these variants with other variants reported in our previous studies. MD simulations were carried out using GROMACS 5.1.3 package [61] with CHARMM27 force field [62]. The protein was enclosed in a cubic box of dimensions 26.9 nm × 26.9 nm × 26.9 nm at least 1.5 nm from the edges. The system was minimized for 50,000 steps using steepest descent algorithm, with an energy step size of 0.01 and a maximum force lower than 1000 kJ/mol/nm. A neighbor list and long-range forces were updated at a frequency of 1 step, using the grid method to determine the neighbor list. The short-range cut-off for making the neighbor list was set to 1.0 nm for electrostatic and Van der Waals interactions. Periodic boundary conditions (PBC) were applied in all three dimensions. MD production run was carried out for 10 ns under microcanonical ensemble (NVE) ensemble at 298 K in the vacuum phase. The simulation was run for 10,000,000 steps with a time step of 1 fs using the leap-frog integrator. The simulation output was saved every 1 ps, including the coordinates, velocities and energies. A total of 10,000 MD trajectories were generated and saved during the simulation. Short-range electrostatic and van der Waals cutoffs were set to 1.0 nm. Bond lengths were not constrained. Long-range electrostatics were computed using particle mesh Ewald method [63] with a cubic interpolation and a grid spacing of 0.16 for the fast Fourier transform (FFT). The Verlet cutoff scheme with a grid-based approach was used for neighbor-searching. The neighbor list was updated every 20 fs. PBC were used for the MD run. Temperature coupling and pressure coupling were turned off. Velocity generation was accomplished by assigning velocities from a Maxwell distribution at a temperature of 298 K, with a random seed. Dispersion correction was applied to account for the cut-off van der Waals scheme. The root-mean-square deviation (RMSD) of the protein backbone atoms with respect to the starting structure was calculated using the ‘gmx rms’ utility within GROMACS [64].

### 4.5. Binding Free Energy Calculation

The binding free energy between full-length SARS-CoV-2 S protein and ACE2 complexes were estimated with MM/PBSA calculations [65] using ‘g_mmpbsa’ tool [66]. Since the MD simulations were performed in a vacuum environment, the MM/PBSA calculations were also conducted under the same vacuum conditions. The ‘g_mmpbsa’ tool is a freely available custom implementation designed to calculate the binding free energy of biomolecular associations. It is a command-line interface that can be executed from a terminal or console with specific options. The ‘g_mmpbsa’ tool was utilized for binding free energy calculations without any additional modifications or customized parameters. An index file was created for the S protein–ACE2 complex by selecting all chains of the S protein (A, B and C) and ACE (I, II and III) using the ‘gmx make_ndx’ module. The index file (.ndx) and MD trajectory files (.xtc and .tpr) in their original file formats were directly used as inputs. The input files were provided through the command line to calculate the molecular mechanics vacuum energy using default options. The resulting output files contained the calculated energies including van der Waals (kJ/mol), electrostatic (kJ/mol) and total energy (kJ/mol). Unlike only RBD-based calculations, all atom interaction energies were computed between the whole domains of the full-length S protein and ACE2 for all variants. The binding free energy (kJ/mol) was calculated between A–I chains for the one-open-complex form, A–I and B–II chains for the two-open-complex form and A–I, B–II and C–III chains for the three-open complex form (Figure 4). MM/PBSA calculations were performed at 10 ps intervals to avoid long computing time. Additionally, the contribution of the residues to the binding free energy was calculated using the ‘decomp’ command line option. This option in the ‘g_mmpbsa’ tool is used to perform decomposition analysis in MM/PBSA calculations. It allows for the breakdown of the overall binding free energy into individual energy contributions from different protein residues. This analysis provides insights into the specific contributions of each residue to the overall binding free energy and can aid in understanding the key interactions driving the binding process.

### 4.6. Distance Calculation

The V503 and N501 residues are located within the RBD region of the S protein and play a crucial role in the binding interface between the S protein and ACE2. These residues exhibit a symmetrical arrangement among the trimeric chains in both the closed (PDB 6VXX) [47] and open (PDB 7A98) [48] forms of the S protein, respectively (Figure 5). To analyze the conformational changes in trimeric S protein chains, we calculated the distances and SD for the distances between V503 residues and between N501 residues across the trimeric chains. The N501 and V503 residues in trimeric S protein chains were selected with ‘gmx make_ndx’ module. The distances (nm) between V503 residues and between N501 residues among A–B, A–C and B–C chains of the S protein were calculated for each MD trajectory using ‘gmx mindist’ module for all variants. This module calculates the minimum distance between two residues in an MD simulation. It measures the closest distance between any atom in one residue and any atom in the other residue over the course of the simulation. SDs (nm) between V503 residues and between N501 residues were calculated from distance values between A–B and A–C, A–B and B–C, A–C and B–C, and A–B–C chains. Distance and SD values were calculated using all 10,000 MD trajectories. These calculations were performed independently for each trajectory to obtain trajectory-specific distances and SDs.

### 4.7. Interaction Analysis 

The intramolecular interactions of SARS-CoV-2 S protein were analyzed in all MD trajectories using Visual Molecular Dynamics (VMD) version 1.9.4 [67]. Hydrogen bond analysis was performed on the entire S protein structure with ‘Hydrogen Bonds’ plugin of VMD, where the definition of a hydrogen bond was the requirement of donor-acceptor atoms being polar, a distance cutoff of 3.5 Å and an angle cutoff of 30°. The definition of hydrogen bonds followed the standard Luzar and Chandler criteria [68]. Hydrogen bond occupancy over the entire simulation time was calculated based on the percentage of MD trajectories in which a given hydrogen bond was present. Salt–bridge interactions were analyzed with ‘Salt Bridges’ plugin of VMD. Salt–bridge interactions were defined as interactions between any of the oxygen atoms of acidic residues and the nitrogen atoms of basic residues within a distance cut-off of 4 Å.

## 5. Conclusions

We used computational methods to analyze the structural stability of the S protein in recent SARS-CoV-2 Omicron subvariants. Differences in stability among Omicron subvariants may have implications for viral entry, infection and antibody evasion. Our results suggest that all Omicron subvariants possess higher levels of stability compared to the wild type. This indicates that mutations in the S protein affect its stability and binding affinity, leading to alterations in viral infectivity and the potential for antibody evasion. Furthermore, the stability and binding affinity of subvariants possibly differs due to variations in the mutations present in the S protein. The identification of more stable subvariants such as BA.2.75, BA.4/BA.5 and BA.2.12.1 and less stable variants such as BA.4.6_N658S, BA.2.75_K147E and BA.4.6 may guide the design of more effective drugs or vaccines that specifically target these variants. Additionally, this study highlights the importance of analyzing the full-length S protein–ACE2 complex rather than just the RBD–ACE2 complex to gain a comprehensive understanding of structural stability. The findings of this study have significant implications for future studies on SARS-CoV-2. The significance of the findings lies in their potential to inform the development of effective therapies against SARS-CoV-2 variants, which is crucial for managing the ongoing COVID-19 pandemic.

## Figures and Tables

**Table 1:** Summary of the total binding free energy (kJ/mol) between SARS-CoV-2 S protein and ACE2 in one-, two- and three-open-complex forms in the final MD trajectory (10 ns). The average total binding free energy (ΔG, kJ/mol) represents the mean value across all (one-, two-, and three-open-complex) forms.

Variant Type	Total Binding Energy			Average Total Binding Energy
	One-Open- Complex Form	Two-Open- Complex Form	Three-Open- Complex Form	All Complex Forms
Wild	−11,773.3	−20,757.0	−17,384.2	−16,638.2
BA.1	−16,980.7	−37,287.8	−45,273.1	−33,180.5
BA.2	−15,130.2	−37,202.6	−45,986.4	−32,773.1
BA.2.12.1	−16,047.9	−34,681.3	−47,719.7	−32,816.3
BA.4/BA.5	−16,364.2	−36,144.4	−47,427.2	−33,311.9
BA.2.75	−18,635.3	−42,068.4	−58,801.1	−39,834.9
BA.2.75_K147E	−14,639.9	−31,729.8	−47,497.0	−31,288.9
BA.4.6	−14,997.9	−31,413.1	−45,491.2	−30,634.1
BA.4.6_N658S	−14,704.4	−29,389.6	−39,351.5	−27,815.2

**Table 2:** Summary of distance and SD values between V503 residues (nm) observed in the final MD trajectory (10 ns).

PDB	Variant Type	A-B	A-C	B-C	SD (A-B) & (A-C)	SD (A-B) & (B-C)	SD (A-C) & (B-C)	SD (A-B-C)
One-open- complex form	Wild	3.00	3.01	0.25	0.01	1.95	1.95	1.59
	BA.1	3.33	3.28	0.91	0.04	1.72	1.68	1.38
	BA.2	2.42	2.37	0.28	0.03	1.51	1.48	1.22
	BA.2.12.1	2.61	2.18	0.19	0.31	1.71	1.41	1.29
	BA.4/BA.5	2.35	2.12	0.26	0.16	1.48	1.32	1.15
	BA.2.75	3.00	2.99	0.43	0.01	1.82	1.81	1.48
	BA.2.75_K147E	2.47	1.08	1.01	0.98	1.03	0.05	0.82
	BA.4.6	2.77	2.29	0.30	0.34	1.75	1.40	1.31
	BA.4.6_N658S	2.92	2.89	0.29	0.02	1.86	1.84	1.51
Two-open- complex form	Wild	13.96	3.57	10.63	7.35	2.36	5.00	5.31
	BA.1	9.81	3.34	6.73	4.57	2.18	2.40	3.24
	BA.2	9.67	3.28	7.81	4.52	1.32	3.20	3.29
	BA.2.12.1	7.42	3.40	5.81	2.84	1.14	1.70	2.02
	BA.4/BA.5	7.77	3.16	5.67	3.26	1.48	1.77	2.31
	BA.2.75	9.78	2.57	7.50	5.10	1.62	3.48	3.69
	BA.2.75_K147E	9.31	3.15	6.90	4.35	1.70	2.65	3.10
	BA.4.6	7.88	3.53	4.86	3.07	2.14	0.94	2.23
	BA.4.6_N658S	8.98	2.87	6.05	4.32	2.07	2.25	3.06
Three-open- complex form	Wild	8.23	10.32	9.75	1.48	1.08	0.40	1.08
	BA.1	10.32	8.81	9.89	1.06	0.31	0.76	0.78
	BA.2	8.87	8.48	7.56	0.27	0.93	0.65	0.67
	BA.2.12.1	9.19	8.88	8.53	0.22	0.46	0.24	0.33
	BA.4/BA.5	8.53	9.39	8.40	0.60	0.09	0.69	0.53
	BA.2.75	8.89	7.75	8.36	0.81	0.38	0.43	0.57
	BA.2.75_K147E	11.10	8.70	8.95	1.69	1.52	0.18	1.32
	BA.4.6	11.45	8.73	9.77	1.93	1.19	0.73	1.37
	BA.4.6_N658S	7.81	9.81	9.64	1.41	1.29	0.12	1.11

**Table 3:** Summary of distance and SD values between N501 residues (nm) observed in the final MD trajectory (10 ns).

PDB	Variant Type	A-B	A-C	B-C	SD (A-B) & (A-C)	SD (A-B) & (B-C)	SD (A-C) & (B-C)	SD (A-B-C)
One-open- complex form	Wild	3.33	2.91	0.43	0.30	2.05	1.75	1.57
	BA.1	3.57	2.85	1.16	0.51	1.71	1.19	1.24
	BA.2	2.99	2.06	0.56	0.66	1.72	1.06	1.23
	BA.2.12.1	3.01	1.42	0.98	1.13	1.44	0.31	1.07
	BA.4/BA.5	2.56	1.75	0.62	0.57	1.37	0.80	0.98
	BA.2.75	2.98	2.08	0.81	0.64	1.53	0.89	1.09
	BA.2.75_K147E	3.36	1.47	1.67	1.33	1.19	0.14	1.04
	BA.4.6	2.95	2.33	0.70	0.44	1.59	1.15	1.16
	BA.4.6_N658S	2.81	2.12	0.48	0.49	1.65	1.16	1.20
Two-open- complex form	Wild	13.93	3.10	11.08	7.66	2.02	5.64	4.66
	BA.1	8.99	3.02	6.57	4.22	1.71	2.50	3.00
	BA.2	10.71	2.96	8.98	5.48	1.23	4.26	4.07
	BA.2.12.1	8.08	2.96	6.50	3.62	1.11	2.51	2.62
	BA.4/BA.5	8.62	2.87	6.65	4.07	1.39	2.68	2.92
	BA.2.75	10.85	2.50	8.83	5.90	1.43	4.47	4.35
	BA.2.75_K147E	10.07	2.55	8.23	5.31	1.30	4.02	3.92
	BA.4.6	8.64	2.81	6.27	4.12	1.68	2.45	2.93
	BA.4.6_N658S	9.11	2.89	6.59	4.40	1.78	2.62	3.13
Three-open- complex form	Wild	8.04	10.29	9.56	1.59	1.07	0.52	1.15
	BA.1	9.71	8.98	9.69	0.52	0.02	0.50	0.42
	BA.2	9.72	9.14	8.72	0.41	0.71	0.30	0.50
	BA.2.12.1	10.44	10.01	9.25	0.30	0.84	0.54	0.60
	BA.4/BA.5	9.86	9.97	9.31	0.07	0.39	0.47	0.36
	BA.2.75	9.42	8.74	9.53	0.48	0.08	0.56	0.43
	BA.2.75_K147E	11.82	9.69	10.17	1.51	1.17	0.34	1.12
	BA.4.6	12.60	9.66	11.01	2.08	1.12	0.95	1.47
	BA.4.6_N658S	7.99	10.73	9.21	1.94	0.87	1.07	1.37

## Data Availability

The SARS-CoV-2 whole-genome sequences described are available in GISAID (Accession IDs EPI_ISL_8885887, 13086512, 13086514, 13086515, 13086516, 14507613, and 14780352).

## Supplementary Materials

The following supporting information can be downloaded at: https://www.mdpi.com/article/10.3390/ijms242216069/s1.

## References

[1] Kumar V., Singh J., Hasnain S.E., Sundar D. (2021). Possible link between higher transmissibility of alpha, kappa and delta variants of SARS-CoV-2 and increased structural stability of its spike protein and hACE2 affinity. Int. J. Mol. Sci..

[2] Ou J., Zhou Z., Dai R., Zhang J., Zhao S., Wu X., Lan W., Ren Y., Cui L., Lan Q. (2021). V367F mutation in SARS-CoV-2 spike RBD emerging during the early transmission phase enhances viral infectivity through increased human ACE2 receptor binding affinity. J. Virol..

[3] Mahmoudi Gomari M., Rostami N., Omidi-Ardali H., Arab S.S. (2022). Insight into molecular characteristics of SARS-CoV-2 spike protein following D614G point mutation, a molecular dynamics study. J. Biomol. Struct. Dyn..

[4] Idowu K.A., Onyenaka C., Olaleye O.A. (2022). A computational evaluation of structural stability of omicron and delta mutations of SARS-CoV-2 spike proteins and human ACE-2 interactions. Inform. Med. Unlocked.

[5] Bazargan M., Elahi R., Esmaeilzadeh A. (2022). OMICRON: Virology, immunopathogenesis, and laboratory diagnosis. J. Gene Med..

[6] Ghosh N., Nandi S., Saha I. (2022). A review on evolution of emerging SARS-CoV-2 variants based on spike glycoprotein. Int. Immunopharmacol..

[7] Hossain A., Akter S., Rashid A.A., Khair S., Alam A.R.U. (2022). Unique mutations in SARS-CoV-2 Omicron subvariants’ non-spike proteins: Potential impacts on viral pathogenesis and host immune evasion. Microb. Pathog..

[8] Ke H., Chang M.R., Marasco W.A. (2022). Immune evasion of SARS-CoV-2 omicron subvariants. Vaccines.

[9] Kumar S., Karuppanan K., Subramaniam G. (2022). Omicron (BA. 1) and sub-variants (BA. 1.1, BA. 2, and BA. 3) of SARS-CoV-2 spike infectivity and pathogenicity: A comparative sequence and structural-based computational assessment. J. Med. Virol..

[10] Ou J., Lan W., Wu X., Zhao T., Duan B., Yang P., Ren Y., Quan L., Zhao W., Seto D. (2022). Tracking SARS-CoV-2 Omicron diverse spike gene mutations identifies multiple inter-variant recombination events. Signal Transduct. Target. Ther..

[11] Cao Y., Song W., Wang L., Liu P., Yue C., Jian F., Yu Y., Yisimayi A., Wang P., Wang Y. (2022). Characterization of the enhanced infectivity and antibody evasion of Omicron BA. 2.75. Cell Host Microbe.

[12] Huo J., Dijokaite-Guraliuc A., Liu C., Zhou D., Ginn H.M., Das R., Supasa P., Selvaraj M., Nutalai R., Tuekprakhon A. (2023). A delicate balance between antibody evasion and ACE2 affinity for Omicron BA. 2.75. Cell Rep..

[13] Cao Y., Yisimayi A., Jian F., Song W., Xiao T., Wang L., Du S., Wang J., Li Q., Chen X. (2022). BA. 2.12. 1, BA. 4 and BA. 5 escape antibodies elicited by Omicron infection. Nature.

[14] Wang Q., Guo Y., Iketani S., Nair M.S., Li Z., Mohri H., Wang M., Yu J., Bowen A.D., Chang J.Y. (2022). Antibody evasion by SARS-CoV-2 Omicron subvariants BA. 2.12. 1, BA. 4 and BA. 5. Nature.

[15] Tuekprakhon A., Nutalai R., Dijokaite-Guraliuc A., Zhou D., Ginn H.M., Selvaraj M., Liu C., Mentzer A.J., Supasa P., Duyvesteyn H.M. (2022). Antibody escape of SARS-CoV-2 Omicron BA. 4 and BA. 5 from vaccine and BA. 1 serum. Cell.

[16] Dejnirattisai W., Huo J., Zhou D., Zahradník J., Supasa P., Liu C., Duyvesteyn H.M., Ginn H.M., Mentzer A.J., Tuekprakhon A. (2022). SARS-CoV-2 Omicron-B. 1.1. 529 leads to widespread escape from neutralizing antibody responses. Cell.

[17] Nutalai R., Zhou D., Tuekprakhon A., Ginn H.M., Supasa P., Liu C., Huo J., Mentzer A.J., Duyvesteyn H.M., Dijokaite-Guraliuc A. (2022). Potent cross-reactive antibodies following Omicron breakthrough in vaccinees. Cell.

[18] Kim S., Liu Y., Ziarnik M., Seo S., Cao Y., Zhang X.F., Im W. (2023). Binding of human ACE2 and RBD of omicron enhanced by unique interaction patterns among SARS-CoV-2 variants of concern. J. Comput. Chem..

[19] Abas A.H., Marfuah S., Idroes R., Kusumawaty D., Fatimawali, Park M.N., Siyadatpanah A., Alhumaydhi F.A., Mahmud S., Tallei T.E. (2022). Can the SARS-CoV-2 omicron variant confer natural immunity against COVID-19?. Molecules.

[20] Tallei T.E., Alhumaid S., AlMusa Z., Fatimawali, Kusumawaty D., Alynbiawi A., Alshukairi A.N., Rabaan A.A. (2023). Update on the omicron sub-variants BA. 4 and BA. 5. Rev. Med. Virol..

[21] Rahmani A.H., Anwar S., Raut R., Almatroudi A., Babiker A.Y., Khan A.A., Alsahli M.A., Almatroodi S.A. (2022). Therapeutic potential of myrrh, a natural resin, in health management through modulation of oxidative stress, inflammation, and advanced glycation end products formation using in vitro and in silico analysis. Appl. Sci..

[22] Dhama K., Nainu F., Frediansyah A., Yatoo M.I., Mohapatra R.K., Chakraborty S., Zhou H., Islam M.R., Mamada S.S., Kusuma H.I. (2023). Global emerging Omicron variant of SARS-CoV-2: Impacts, challenges and strategies. J. Infect. Public Health.

[23] Fan Y., Li X., Zhang L., Wan S., Zhang L., Zhou F. (2022). SARS-CoV-2 Omicron variant: Recent progress and future perspectives. Signal Transduct. Target. Ther..

[24] Silva S.J.R.d., Kohl A., Pena L., Pardee K. (2023). Recent insights into SARS-CoV-2 omicron variant. Rev. Med. Virol..

[25] Chatterjee S., Bhattacharya M., Nag S., Dhama K., Chakraborty C. (2023). A detailed overview of SARS-CoV-2 omicron: Its sub-variants, mutations and pathophysiology, clinical characteristics, immunological landscape, immune escape, and therapies. Viruses.

[26] Dhawan M., Saied A.A., Mitra S., Alhumaydhi F.A., Emran T.B., Wilairatana P. (2022). Omicron variant (B. 1.1. 529) and its sublineages: What do we know so far amid the emergence of recombinant variants of SARS-CoV-2?. Biomed. Pharmacother..

[27] Shrestha L.B., Foster C., Rawlinson W., Tedla N., Bull R.A. (2022). Evolution of the SARS-CoV-2 omicron variants BA. 1 to BA. 5: Implications for immune escape and transmission. Rev. Med. Virol..

[28] Xu A., Hong B., Lou F., Wang S., Li W., Shafqat A., An X., Zhao Y., Song L., Tong Y. (2022). Sub-lineages of the SARS-CoV-2 Omicron variants: Characteristics and prevention. MedComm.

[29] Hachmann N.P., Miller J., Collier A.-r.Y., Barouch D.H. (2022). Neutralization escape by SARS-CoV-2 Omicron subvariant BA. 4.6. N. Engl. J. Med..

[30] Hachmann N.P., Miller J., Collier A.-r.Y., Ventura J.D., Yu J., Rowe M., Bondzie E.A., Powers O., Surve N., Hall K. (2022). Neutralization escape by SARS-CoV-2 Omicron subvariants BA. 2.12. 1, BA. 4, and BA. 5. N. Engl. J. Med..

[31] Kimura I., Yamasoba D., Tamura T., Nao N., Suzuki T., Oda Y., Mitoma S., Ito J., Nasser H., Zahradnik J. (2022). Virological characteristics of the SARS-CoV-2 Omicron BA. 2 subvariants, including BA. 4 and BA. 5. Cell.

[32] Pastorio C., Zech F., Noettger S., Jung C., Jacob T., Sanderson T., Sparrer K.M., Kirchhoff F. (2022). Determinants of Spike infectivity, processing, and neutralization in SARS-CoV-2 Omicron subvariants BA. 1 and BA. 2. Cell Host Microbe.

[33] Tan C.-W., Lim B.-L., Young B.E., Yeoh A.Y.-Y., Yung C.-F., Yap W.-C., Althaus T., Chia W.-N., Zhu F., Lye D.C. (2022). Comparative neutralisation profile of SARS-CoV-2 omicron subvariants BA. 2.75 and BA. 5. Lancet Microbe.

[34] Tian D., Nie W., Sun Y., Ye Q. (2022). The epidemiological features of the SARS-CoV-2 Omicron subvariant BA. 5 and its evasion of the neutralizing activity of vaccination and prior infection. Vaccines.

[35] Yamasoba D., Kosugi Y., Kimura I., Fujita S., Uriu K., Ito J., Sato K. (2022). Neutralisation sensitivity of SARS-CoV-2 omicron subvariants to therapeutic monoclonal antibodies. Lancet Infect. Dis..

[36] Choudhury A., Mukherjee S. (2020). In silico studies on the comparative characterization of the interactions of SARS-CoV-2 spike glycoprotein with ACE-2 receptor homologs and human TLRs. J. Med. Virol..

[37] Choudhury A., Das N.C., Patra R., Mukherjee S. (2021). In silico analyses on the comparative sensing of SARS-CoV-2 mRNA by the intracellular TLRs of humans. J. Med. Virol..

[38] Choudhury A., Mukherjee G., Mukherjee S. (2021). Chemotherapy vs. Immunotherapy in combating nCOVID19: An update. Hum. Immunol..

[39] Choi K.-E., Kim J.-M., Rhee J., Park A.K., Kim E.-J., Kang N.S. (2021). Molecular dynamics studies on the structural characteristics for the stability prediction of SARS-CoV-2. Int. J. Mol. Sci..

[40] Choi K.-E., Kim J.-M., Rhee J.E., Park A.K., Kim E.-J., Yoo C.K., Kang N.S. (2022). Molecular dynamics studies on the structural stability prediction of SARS-CoV-2 variants including multiple mutants. Int. J. Mol. Sci..

[41] Singh J., Anantharaj A., Panwar A., Rani C., Bhardwaj M., Kumar P., Chattopadhyay P., Devi P., Maurya R., Mishra P. (2023). BA. 1, BA. 2 and BA. 2.75 variants show comparable replication kinetics, reduced impact on epithelial barrier and elicit cross-neutralizing antibodies. PLoS Pathog..

[42] Gupta A.M., Chakrabarti J. (2023). Effect on the conformations of the spike protein of SARS-CoV-2 due to mutation. Biotechnol. Appl. Biochem..

[43] Akbulut E. (2021). Mutations in the SARS-CoV-2 spike protein may cause functional changes in the protein quaternary structure. Turk. J. Biochem..

[44] Xu Y., Wu C., Cao X., Gu C., Liu H., Jiang M., Wang X., Yuan Q., Wu K., Liu J. (2022). Structural and biochemical mechanism for increased infectivity and immune evasion of Omicron BA. 2 variant compared to BA. 1 and their possible mouse origins. Cell Res..

[45] Lee M., Major M., Hong H. (2023). Distinct conformations of SARS-CoV-2 Omicron spike protein and its interaction with ACE2 and antibody. Int. J. Mol. Sci..

[46] Das N.C., Chakraborty P., Bayry J., Mukherjee S. (2023). Comparative binding ability of human monoclonal antibodies against Omicron variants of SARS-CoV-2: An in silico investigation. Antibodies.

[47] Walls A.C., Park Y.-J., Tortorici M.A., Wall A., McGuire A.T., Veesler D. (2020). Structure, function, and antigenicity of the SARS-CoV-2 spike glycoprotein. Cell.

[48] Benton D.J., Wrobel A.G., Xu P., Roustan C., Martin S.R., Rosenthal P.B., Skehel J.J., Gamblin S.J. (2020). Receptor binding and priming of the spike protein of SARS-CoV-2 for membrane fusion. Nature.

[49] Van Doremalen N., Bushmaker T., Morris D.H., Holbrook M.G., Gamble A., Williamson B.N., Tamin A., Harcourt J.L., Thornburg N.J., Gerber S.I. (2020). Aerosol and surface stability of SARS-CoV-2 as compared with SARS-CoV-1. N. Engl. J. Med..

[50] Nguyen H.L., Thai N.Q., Nguyen P.H., Li M.S. (2022). SARS-CoV-2 omicron variant binds to human cells more strongly than the wild type: Evidence from molecular dynamics simulation. J. Phys. Chem. B.

[51] Lupala C.S., Ye Y., Chen H., Su X.-D., Liu H. (2022). Mutations on RBD of SARS-CoV-2 Omicron variant result in stronger binding to human ACE2 receptor. Biochem. Biophys. Res. Commun..

[52] Celik I., Abdellattif M.H., Tallei T.E. (2022). An insight based on computational analysis of the interaction between the receptor-binding domain of the omicron variants and human angiotensin-converting enzyme 2. Biology.

[53] Lan J., Ge J., Yu J., Shan S., Zhou H., Fan S., Zhang Q., Shi X., Wang Q., Zhang L. (2020). Structure of the SARS-CoV-2 spike receptor-binding domain bound to the ACE2 receptor. Nature.

[54] Kim J.-M., Chung Y.-S., Jo H.J., Lee N.-J., Kim M.S., Woo S.H., Park S., Kim J.W., Kim H.M., Han M.-G. (2020). Identification of coronavirus isolated from a patient in Korea with COVID-19. Osong Public Health Res. Perspect..

[55] Hadfield J., Megill C., Bell S.M., Huddleston J., Potter B., Callender C., Sagulenko P., Bedford T., Neher R.A. (2018). Nextstrain: Real-time tracking of pathogen evolution. Bioinformatics.

[56] Rambaut A., Holmes E.C., O’Toole Á., Hill V., McCrone J.T., Ruis C., du Plessis L., Pybus O.G. (2020). A dynamic nomenclature proposal for SARS-CoV-2 lineages to assist genomic epidemiology. Nat. Microbiol..

[57] Waterhouse A., Bertoni M., Bienert S., Studer G., Tauriello G., Gumienny R., Heer F.T., de Beer T.A.P., Rempfer C., Bordoli L. (2018). SWISS-MODEL: Homology modelling of protein structures and complexes. Nucleic Acids Res..

[58] Camacho C., Coulouris G., Avagyan V., Ma N., Papadopoulos J., Bealer K., Madden T.L. (2009). BLAST+: Architecture and applications. BMC Bioinform..

[59] Steinegger M., Meier M., Mirdita M., Vöhringer H., Haunsberger S.J., Söding J. (2019). HH-suite3 for fast remote homology detection and deep protein annotation. BMC Bioinform..

[60] Studer G., Rempfer C., Waterhouse A.M., Gumienny R., Haas J., Schwede T. (2020). QMEANDisCo—Distance constraints applied on model quality estimation. Bioinformatics.

[61] Abraham M.J., Murtola T., Schulz R., Páll S., Smith J.C., Hess B., Lindahl E. (2015). GROMACS: High performance molecular simulations through multi-level parallelism from laptops to supercomputers. SoftwareX.

[62] Bjelkmar P., Larsson P., Cuendet M.A., Hess B., Lindahl E. (2010). Implementation of the CHARMM force field in GROMACS: Analysis of protein stability effects from correction maps, virtual interaction sites, and water models. J. Chem. Theory Comput..

[63] Darden T., York D., Pedersen L. (1993). Particle mesh Ewald: An N·log (N) method for Ewald sums in large systems. J. Chem. Phys..

[64] Maiorov V.N., Crippen G.M. (1995). Size-independent comparison of protein three-dimensional structures. Proteins.

[65] Homeyer N., Gohlke H. (2012). Free energy calculations by the molecular mechanics Poisson− Boltzmann surface area method. Mol. Inform..

[66] Kumari R., Kumar R., Consortium O.S.D.D., Lynn A. (2014). g_mmpbsa A GROMACS tool for high-throughput MM-PBSA calculations. J. Chem. Inf. Model.

[67] Humphrey W., Dalke A., Schulten K. (1996). VMD: Visual molecular dynamics. J. Mol. Graph..

[68] Luzar A., Chandler D. (1996). Hydrogen-bond kinetics in liquid water. Nature.

