# Supplementary material for: Comparative Computational Analysis of Spike Protein Structural Stability in SARS-CoV-2 Omicron Subvariants
Source: Int J Mol Sci. 2023 Nov 8;24(22):16069. doi: 10.3390/ijms242216069 (PMC10671153; doi:10.3390/ijms242216069)
Supplement: Supplementary file 1 [file ijms-24-16069-s001.zip › ijms-2672082-supplementary.pdf]

Supplementary Information

Comparative Computational Analysis of Spike Protein Structural Stability in SARS-CoV-2 Omicron Subvariants

Anand Balupuri [1,†], Jeong-Min Kim [2,†], Kwang-Eun Choi [1], Jin Sun No [2], Il-Hwan Kim [2], Jee Eun Rhee [2], Eun-Jin Kim [2] and Nam Sook Kang [1,*]

[1] Graduate School of New Drug Discovery and Development, Chungnam National University, 99 Daehak-ro, Yuseong-gu, Daejeon 34134, Republic of Korea; abalupuri@cnu.ac.kr (A.B.); hwendiv@naver.com (K.-E.C.)

[2] Division of Emerging Infectious Diseases, Bureau of Infectious Disease Diagnosis Control, Korea Disease, Control and Prevention Agency, 187 Osongsaengmyeong 2-ro, Osong-eup, Heungdeok-gu, Cheongju-si 28159, Republic of Korea; jmkim97@korea.kr (J.-M.K.); njs2564@korea.kr (J.S.N.); ilhwan98@korea.kr (I.-H.K.); jerhee001@korea.kr (J.E.R.)

* Correspondence: nskang@cnu.ac.kr; Tel.: +82-42-821-8626

† These authors contributed equally to this work.

Table S1. Summary of the binding free energy between SARS-CoV-2 S protein and ACE2 in the one-open-complex form in the final MD trajectory (10 ns). V, E and T represent van der Waals, electrostatic and total energy (kJ/mol), respectively.

**Variant Type**			**A–I chains**
	**V**	**E**	**T**
Wild	−1461.5	−10,311.8	−11,773.3
BA.1	−921.2	−16,059.5	−16,980.7
BA.2	−1055.1	−14,075.1	−15,130.2
BA.2.12.1	−1136.8	−14,911.0	−16,047.9
BA.4/BA.5	−1047.6	−15,316.6	−16,364.2
BA.2.75	−1300.3	−17,335.0	−18,635.3
BA.2.75_K147E	−625.1	−14,014.9	−14,639.9
BA.4.6	−1131.4	−13,866.5	−14,997.9
BA.4.6_N658S	−1206.1	−13,498.3	−14,704.4

Table S2. Summary of the binding free energy between SARS-CoV-2 S protein and ACE2 in the two-open-complex form in the final MD trajectory (10 ns). V, E and T represent van der Waals, electrostatic and total energy (kJ/mol), respectively.

**Variant Type**	**A–I chains**			**B–II chains**			**Total**		
	**V**	**E**	**T**	**V**	**E**	**T**	**V**	**E**	**T**
Wild	−1326.4	−13,200.4	−14,526.7	−677.3	−5553.0	−6230.3	−2003.7	−18,753.4	−20,757.0
BA.1	−1343.9	−16,994.6	−18,338.5	−1215.5	−17,733.8	−18,949.3	−2559.5	−34,728.4	−37,287.8
BA.2	−1465.6	−16,342.4	−17,808.0	−1347.8	−18,046.8	−19,394.6	−2813.4	−34,389.2	−37,202.6
BA.2.12.1	−1270.0	−16,678.6	−17,948.6	−1050.8	−15,681.9	−16,732.7	−2320.7	−32,360.6	−34,681.3
BA.4/BA.5	−1437.5	−17,345.2	−18,782.6	−1281.4	−16,080.4	−17,361.7	−2718.8	−33,425.5	−36,144.4
BA.2.75	−1347.7	−18,963.1	−20,310.8	−1926.7	−19,830.9	−21,757.6	−3274.4	−38,794.0	−42,068.4
BA.2.75_K147E	−1578.8	−15,012.2	−16,591.0	−727.0	−14,411.9	−15,138.9	−2305.8	−29,424.1	−31,729.8
BA.4.6	−1510.1	−14,741.7	−16,251.7	−1063.8	−14,097.7	−15,161.4	−2573.8	−28,839.3	−31,413.1
BA.4.6_N658S	−1305.8	−15,739.9	−17,045.7	−676.5	−11,667.4	−12,343.9	−1982.4	−27,407.3	−29,389.6

Table S3. Summary of the binding free energy between SARS-CoV-2 S protein and ACE2 in the three-open-complex form in the final MD trajectory (10 ns). V, E and T represent van der Waals, electrostatic and total energy (kJ/mol), respectively.

**Variant Type**	**A–I chains**			**B–II chains**			**C–III chains**			**Total**		
	**V**	**E**	**T**	**V**	**E**	**T**	**V**	**E**	**T**	**V**	**E**	**T**
Wild	− 535.4	−5278.5	−5813.8	−527.7	−4872.3	−5400.0	−583.0	−5587.4	−6170.4	−1646.1	−15,738.2	−17,384.2
BA.1	−872.7	−14,757.3	−15,630.0	−570.9	−13,743.9	−14,314.8	−1081.3	−14,247.0	−15,328.3	−2524.9	−42,748.2	−45,273.1
BA.2	−892.0	−12,965.4	−13,857.4	−1334.8	−14,978.4	−16,313.3	−1233.2	−14,582.5	−15,815.7	−3460.0	−42,526.4	−45,986.4
BA.2.12.1	−1078.9	−14,960.8	−16,039.6	−1079.5	−14,751.1	−15,830.6	−1195.2	−14,654.2	−15,849.4	−3353.5	−44,366.1	−47,719.7
BA.4/BA.5	−1076.3	−13,471.1	−14,547.4	−1051.2	−15,203.0	−16,254.2	−1046.4	−15,579.3	−16,625.6	−3173.9	−44,253.4	−47,427.2
BA.2.75	−1046.0	−17,413.5	−18,459.5	−1440.6	−19,058.8	−20,499.4	−1192.5	−18,649.7	−19,842.2	−3679.1	−55,122.0	−58,801.1
BA.2.75_K147E	−1392.1	−14,653.8	−16,045.9	−1112.8	−14,614.4	−15,727.2	−1077.0	−14,646.9	−15,723.9	−3582.0	−43,915.1	−47,497.0
BA.4.6	−1037.8	−14,661.7	−15,699.5	−1113.4	−13,476.9	−14,590.3	−1158.3	−14,043.1	−15,201.4	−3309.5	−42,181.7	−45,491.2
BA.4.6_N658S	−1042.8	−13,045.1	−14,087.9	−815.6	−13,385.7	−14,201.3	−553.9	−10,508.4	−11,062.3	−2412.3	−36,939.3	−39,351.5

Residue	19	24	25	26	27	67	69	70	95	142	143	144	145	147	152	157	210	211	212	213	257	339	346	371	373	375	376	405
**Wild**	T	L	P	P	A	A	H	V	T	G	V	Y	Y	K	W	F	I	N	L	V	G	G	R	S	S	S	T	D
**BA.1**	-	-	-	-	-	V	Δ	Δ	I	D	Δ	Δ	Δ	-	-	-	-	Δ	I	-	-	D	-	L	P	F	-	-
**BA.2**	I	Δ	Δ	Δ	S	-	-	-	-	D	-	-	-	-	-	-	-	-	-	G	-	D	-	F	P	F	A	N
**BA.2.12.1**	I	Δ	Δ	Δ	S	-	-	-	-	D	-	-	-	-	-	-	-	-	-	G	-	D	-	F	P	F	A	N
**BA.4/BA.5**	I	Δ	Δ	Δ	S	-	Δ	Δ	-	D	-	-	-	-	-	-	-	-	-	G	-	D	-	F	P	F	A	N
**BA.2.75**	I	Δ	Δ	Δ	S	-	-	-	-	D	-	-	-	-	R	L	V	-	-	G	S	H	-	F	P	F	A	N
**BA.2.75_K147E**	I	Δ	Δ	Δ	S	-	-	-	-	D	-	-	-	E	R	L	V	-	-	G	S	H	-	F	P	F	A	N
**BA.4.6**	I	Δ	Δ	Δ	S	-	Δ	Δ	-	D	-	-	-	-	-	-	-	-	-	G	-	D	T	F	P	F	A	N
**BA.4.6_N658S**	I	Δ	Δ	Δ	S	-	Δ	Δ	-	D	-	-	-	-	-	-	-	-	-	G	-	D	T	F	P	F	A	N

Residue	408	417	440	446	452	460	477	478	484	486	493	496	498	501	505	547	614	655	658	679	681	704	764	796	856	954	969	981
**Wild**	R	K	N	G	L	N	S	T	E	F	Q	G	Q	N	Y	T	D	H	N	N	P	S	N	D	N	Q	N	L
**BA.1**	-	N	K	S	-	-	N	K	A	-	R	S	R	Y	H	K	G	Y	-	K	H	-	K	Y	K	H	K	F
**BA.2**	S	N	K	-	-	-	N	K	A	-	R	-	R	Y	H	-	G	Y	-	K	H	-	K	Y	-	H	K	-
**BA.2.12.1**	S	N	K	-	Q	-	N	K	A	-	R	-	R	Y	H	-	G	Y	-	K	H	L	K	Y	-	H	K	-
**BA.4/BA.5**	S	N	K	-	R	-	N	K	A	V	-	-	R	Y	H	-	G	Y	-	K	H	-	K	Y	-	H	K	-
**BA.2.75**	S	N	K	S	-	K	N	K	A	-	-	-	R	Y	H	-	G	Y	-	K	H	-	K	Y	-	H	K	-
**BA.2.75_K147E**	S	N	K	S	-	K	N	K	A	-	-	-	R	Y	H	-	G	Y	-	K	H	-	K	Y	-	H	K	-
**BA.4.6**	S	N	K	-	R	-	N	K	A	V	-	-	R	Y	H	-	G	Y	-	K	H	-	K	Y	-	H	K	-
**BA.4.6_N658S**	S	N	K	-	R	-	N	K	A	V	-	-	R	Y	H	-	G	Y	S	K	H	-	K	Y	-	H	K	-

Figure S1. Deletions and mutations observed in the S protein of SARS-CoV-2 Omicron subvariants. The symbol Δ represents deletion while hyphen represents no mutation.

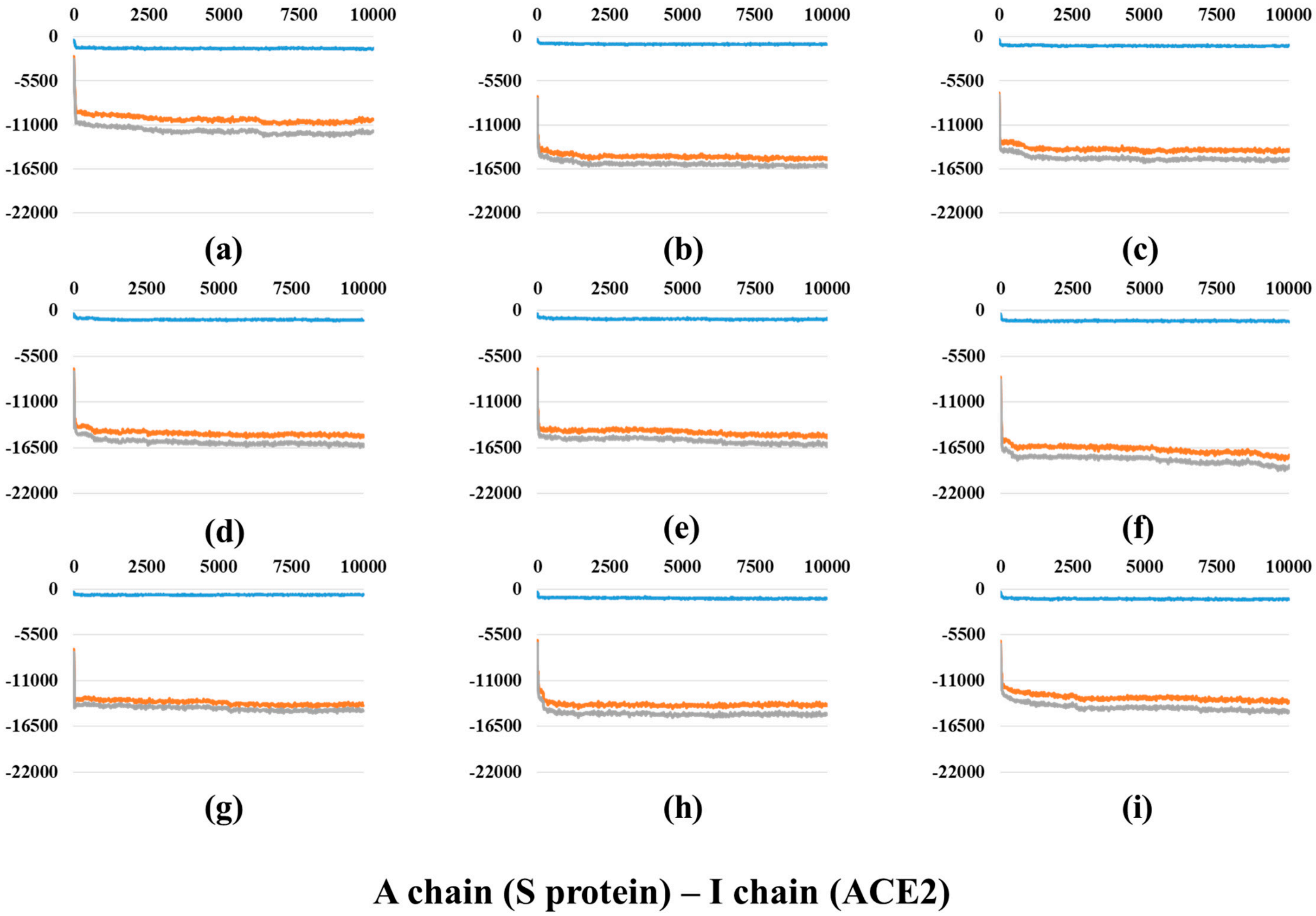

Figure S2. Time evolution of the MM/PBSA energies of A–I chains in the one-open-complex form during 10 ns MD simulation. **(a)** Wild type, **(b)** BA.1, **(c)** BA.2, **(d)** BA.2.12.1, **(e)** BA.4/BA.5, **(f)** BA.2.75, **(g)** BA.2.75_K147E, **(h)** BA.4.6 and **(i)** BA.4.6_N658S. X axis denotes the MD simulation time (ps) and Y axis denotes the MM/PBSA energy value (kJ/mol). Blue color indicates van der Waals energy between S protein and ACE2, and orange color indicates electrostatic energy.

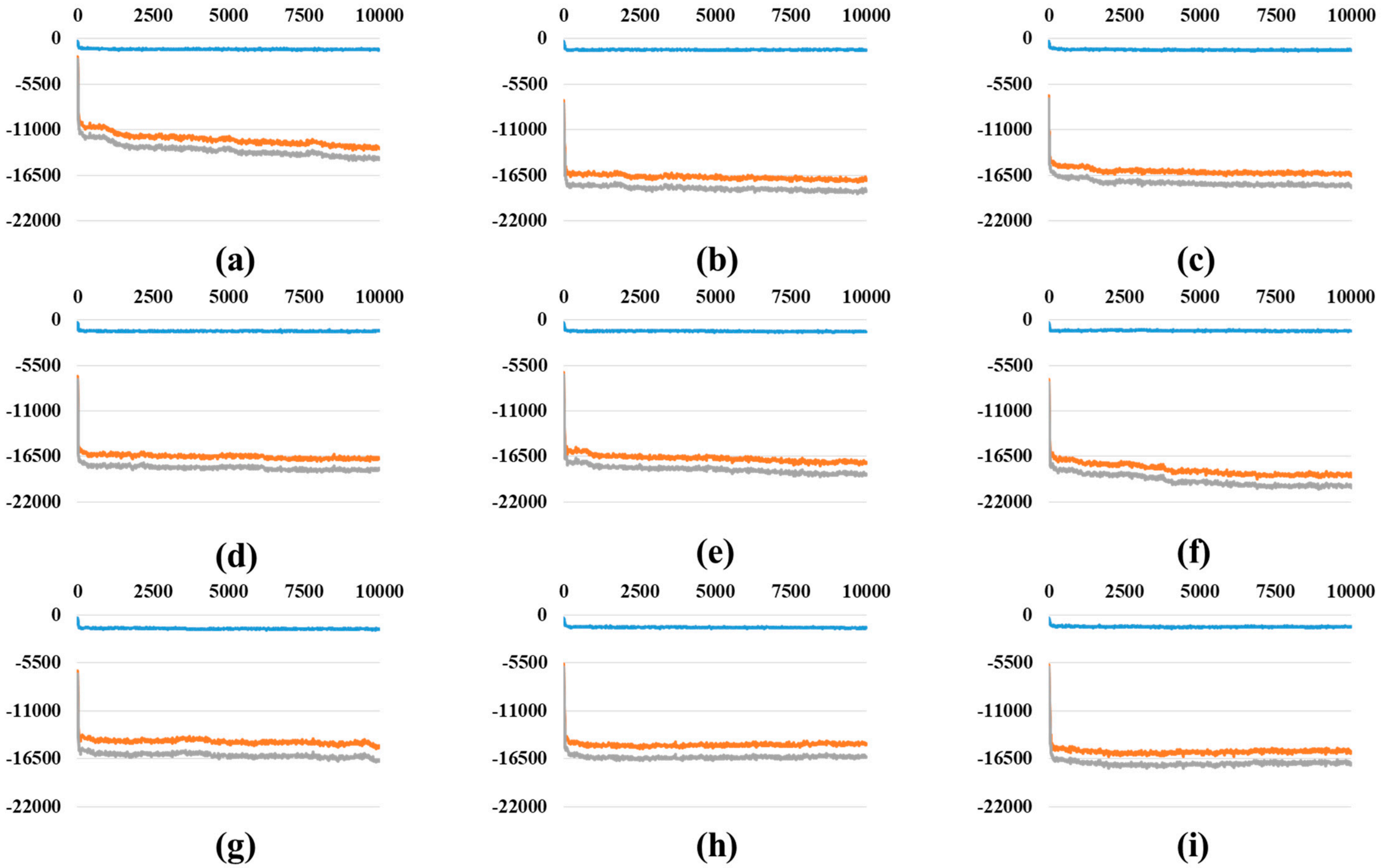

(1) A chain (S protein) – I chain (ACE2)

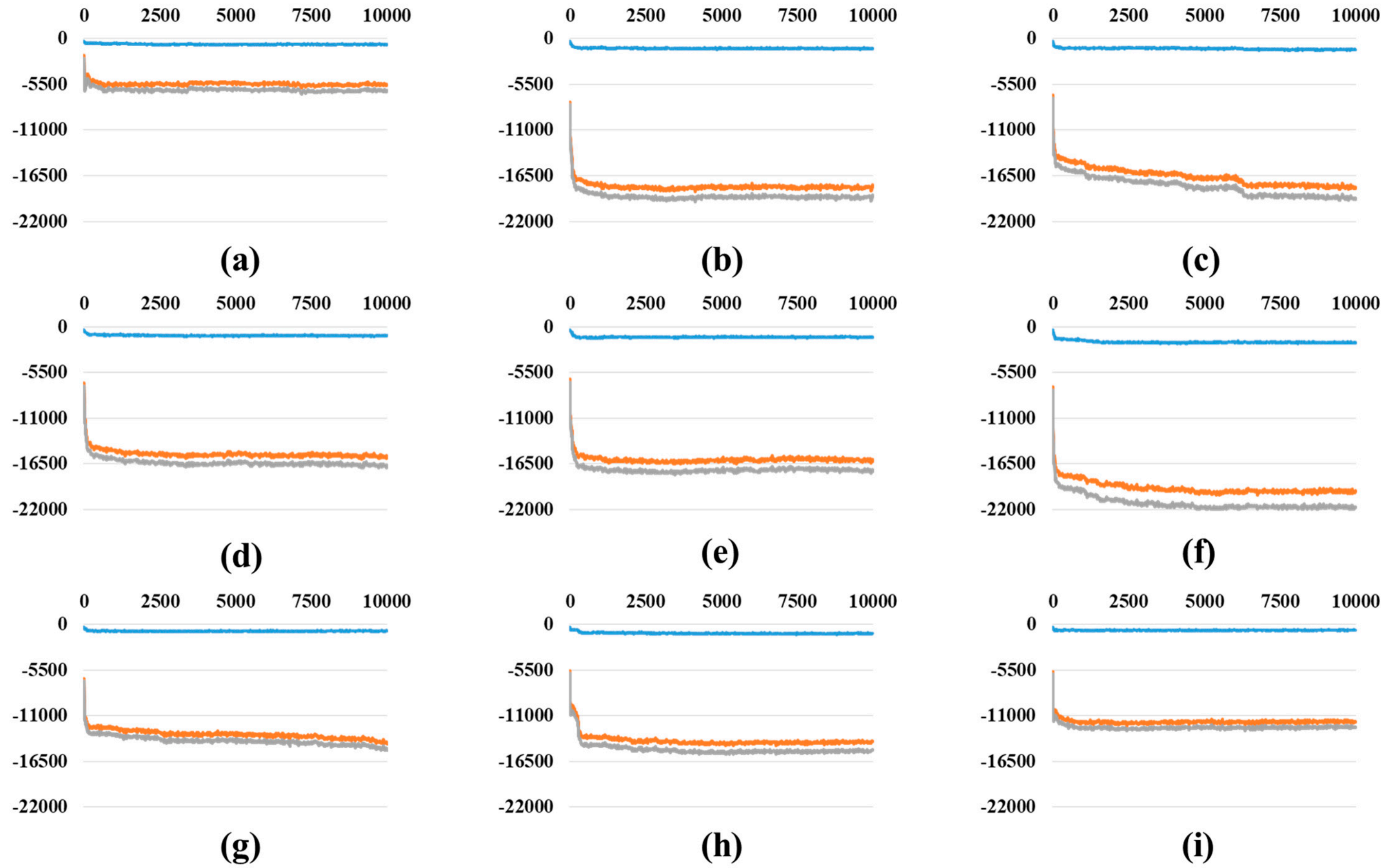

(2) B chain (S protein) – II chain (ACE2)

Figure S3. Time evolution of the MM/PBSA energies of A–I **(1)** and B–II **(2)** chains in the two-open-complex form during 10 ns MD simulation. **(a)** Wild type, **(b)** BA.1, **(c)** BA.2, **(d)** BA.2.12.1, **(e)** BA.4/BA.5, **(f)** BA.2.75, **(g)** BA.2.75_K147E, **(h)** BA.4.6 and **(i)** BA.4.6_N658S. X axis denotes the MD simulation time (ps) and Y axis denotes the MM/PBSA energy value (kJ/mol). Blue color indicates van der Waals energy between S protein and ACE2, and orange color indicates electrostatic energy.

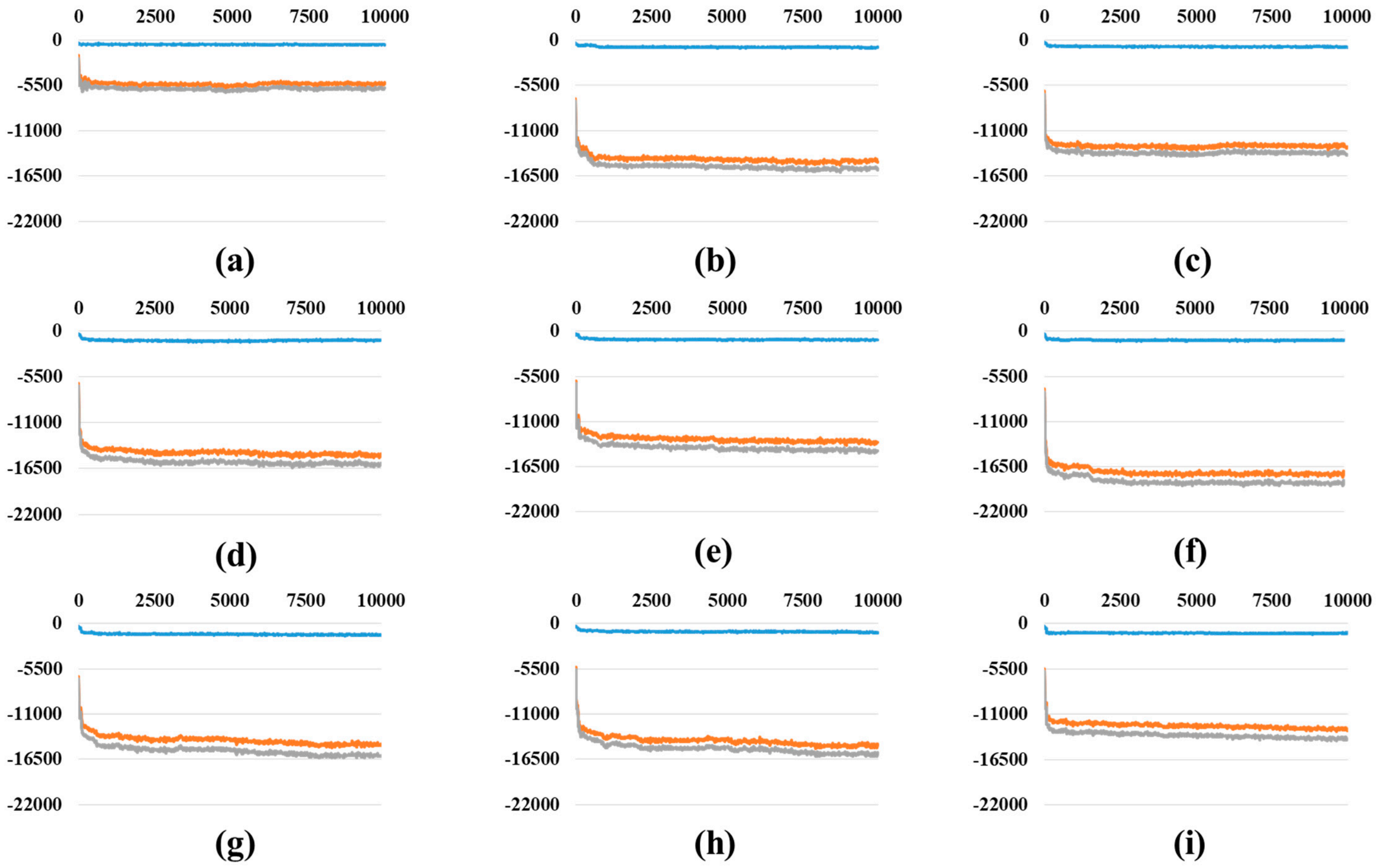

(1) A chain (S protein) – I chain (ACE2)

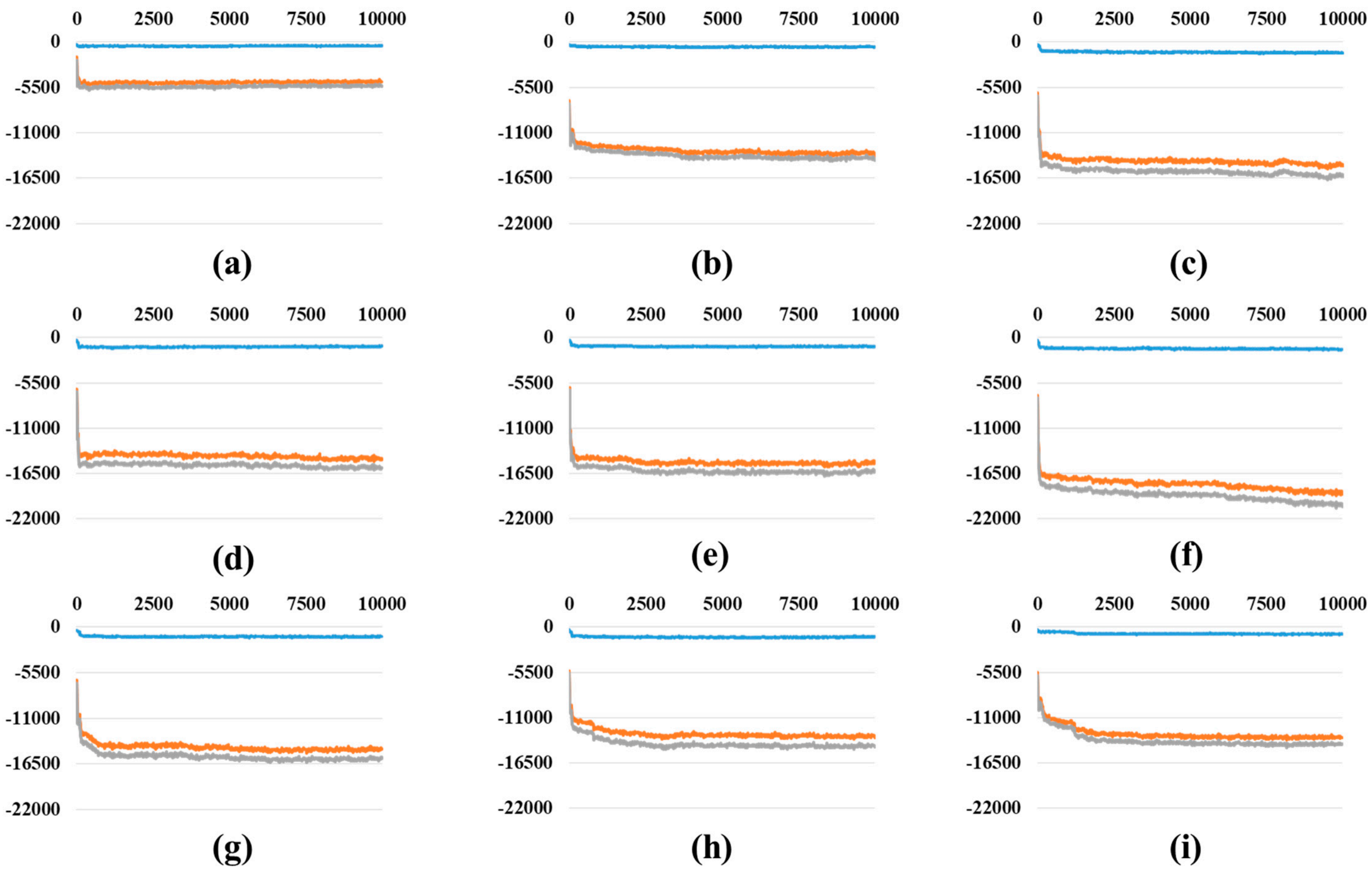

(2) B chain (S protein) – II chain (ACE2)

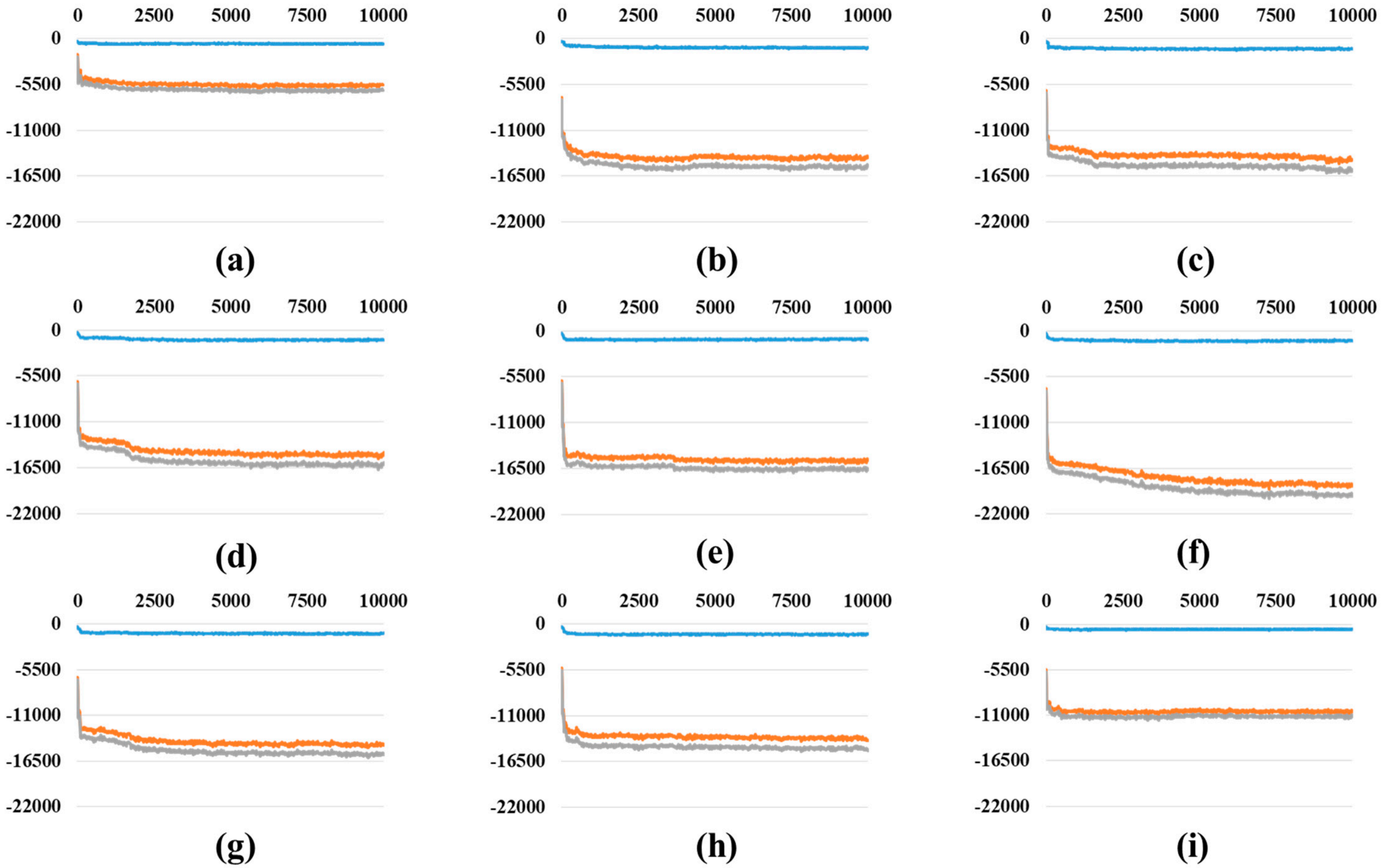

(3) C chain (S protein) – III chain (ACE2)

Figure S4. Time evolution of the MM/PBSA energies of A–I **(1)**, B–II **(2)** and C–III **(3)** chains in the three-open-complex form during 10 ns MD simulation. **(a)** Wild type, **(b)** BA.1, **(c)** BA.2, **(d)** BA.2.12.1, **(e)** BA.4/BA.5, **(f)** BA.2.75, **(g)** BA.2.75_K147E, **(h)** BA.4.6 and **(i)** BA.4.6_N658S. X axis denotes the MD simulation time (ps) and Y axis denotes the MM/PBSA energy value (kJ/mol). Blue color indicates van der Waals energy between S protein and ACE2, and orange color indicates electrostatic energy.

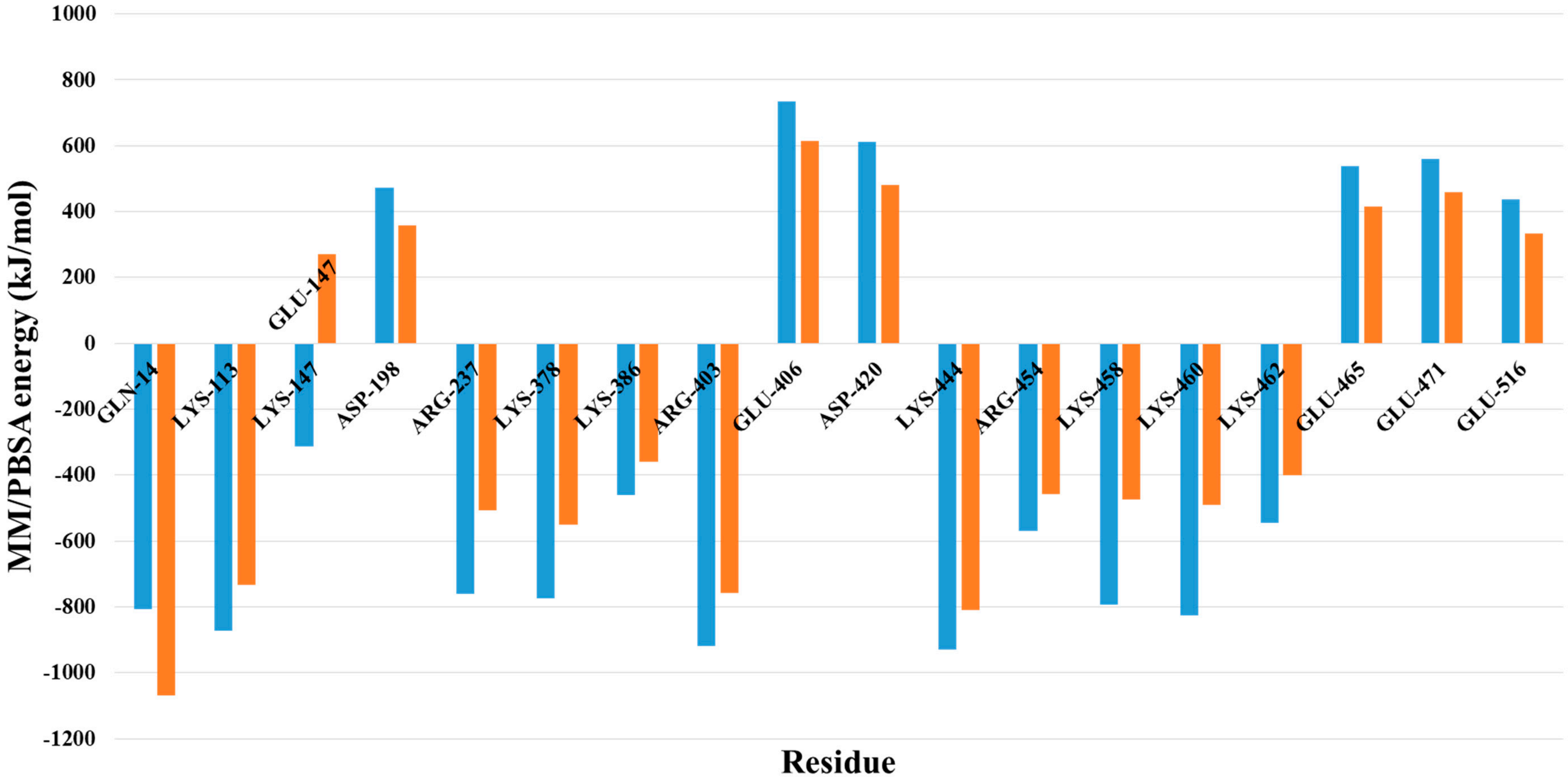

Figure S5. Contribution of residues to the binding free energy in the one-open-complex form. The X axis represents the residues with energy difference more than 100 kJ/mol and the Y axis represents MM/PBSA energy (kJ/mol). Blue indicates the contribution of the BA.2.75 residues to the binding free energy while orange indicates the contribution of the BA.2.75_K147E residues to the binding free energy.

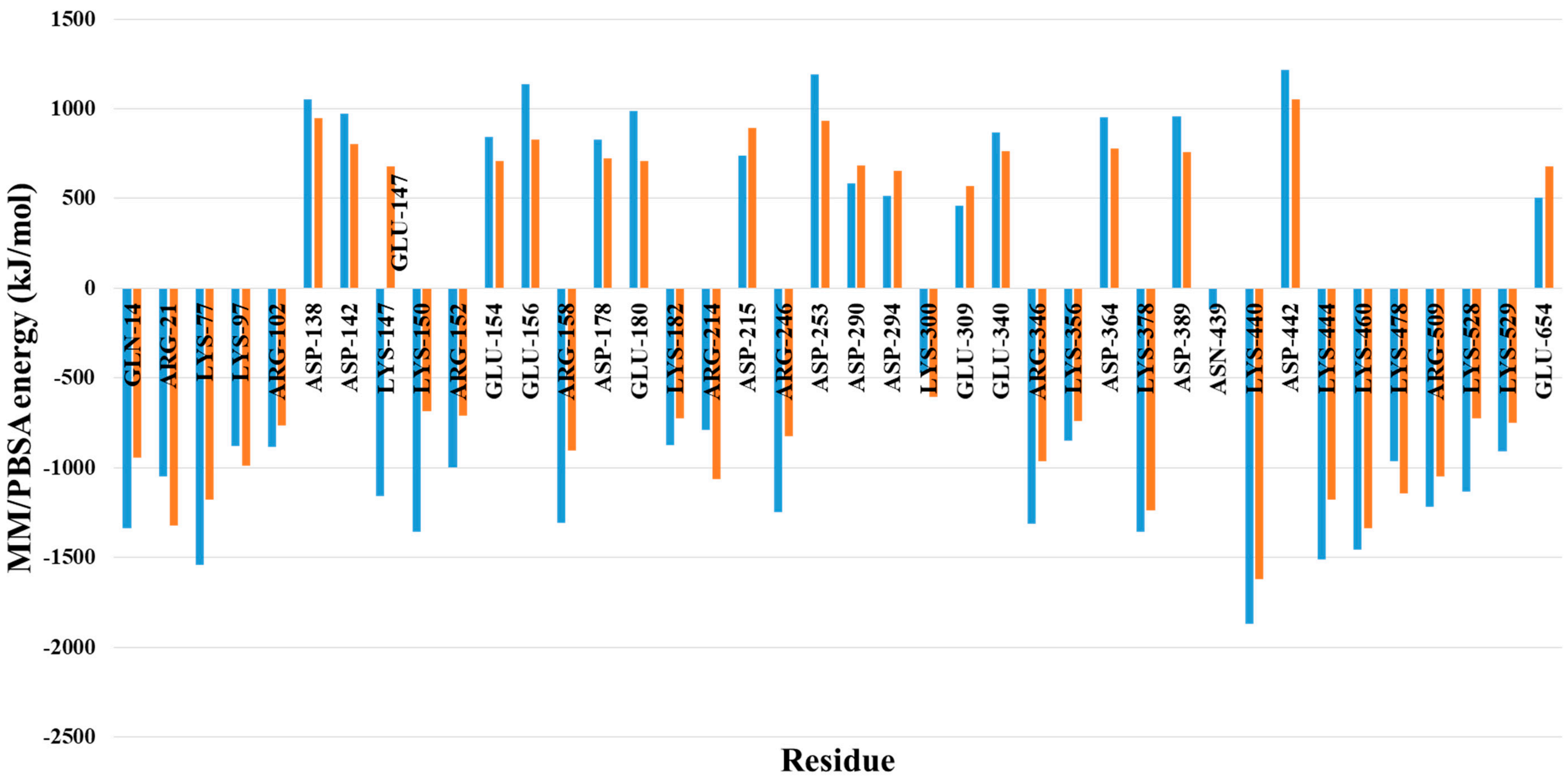

Figure S6. Contribution of residues to the binding free energy in the two-open-complex form. The X axis represents the residues with energy difference more than 100 kJ/mol and the Y axis represents MM/PBSA energy (kJ/mol). Blue indicates the contribution of the BA.2.75 residues to the binding free energy while orange indicates the contribution of the BA.2.75_K147E residues to the binding free energy.

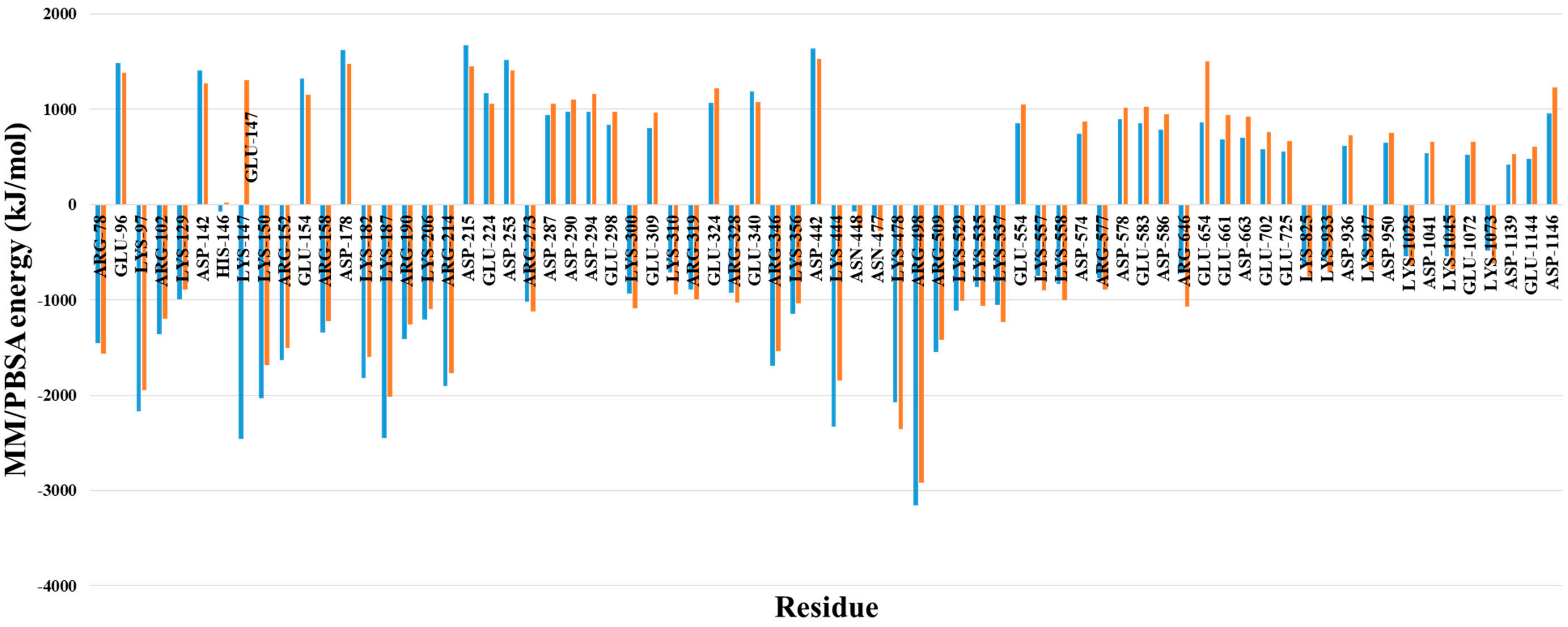

Figure S7. Contribution of residues to the binding free energy in the three-open-complex form. The X axis represents the residues with energy difference more than 100 kJ/mol and the Y axis represents MM/PBSA energy (kJ/mol). Blue indicates the contribution of the BA.2.75 residues to the binding free energy while orange indicates the contribution of the BA.2.75_K147E residues to the binding free energy.

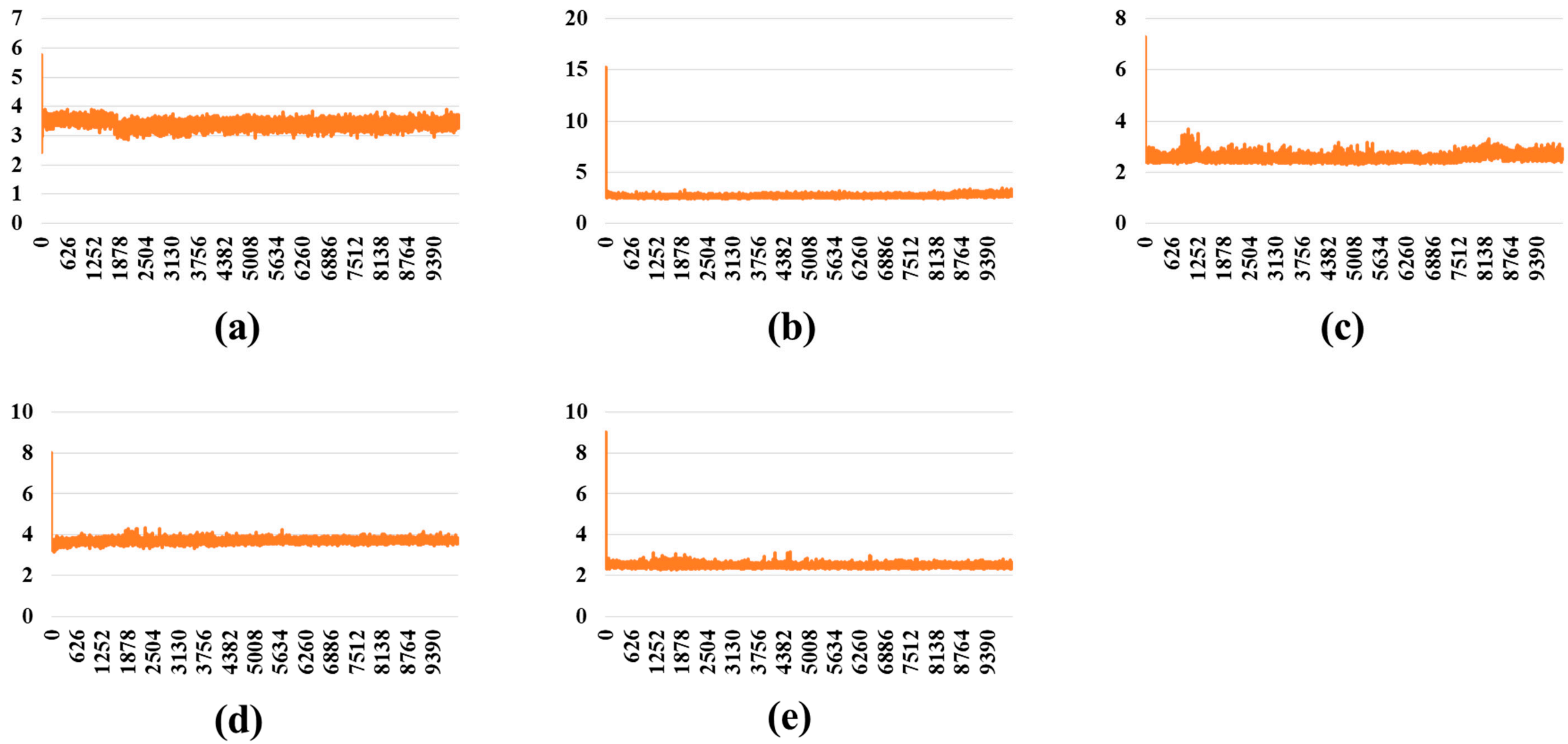

Figure S8. Salt bridge interaction distance between the residue pair in BA.2.75_K147E during 10 ns MD simulation. One-open-complex form (chain A): **(a)** GLU147-LYS150 and **(b)** GLU147-LYS182; two-open-complex form (chain A): **(c)** GLU147-LYS150; and three-open-complex form (chain B): **(d)** GLU147-LYS150 and (chain C): **(e)** GLU147-LYS150. X axis represents the MD simulation time (ps) and Y axis represents the distance (Å).

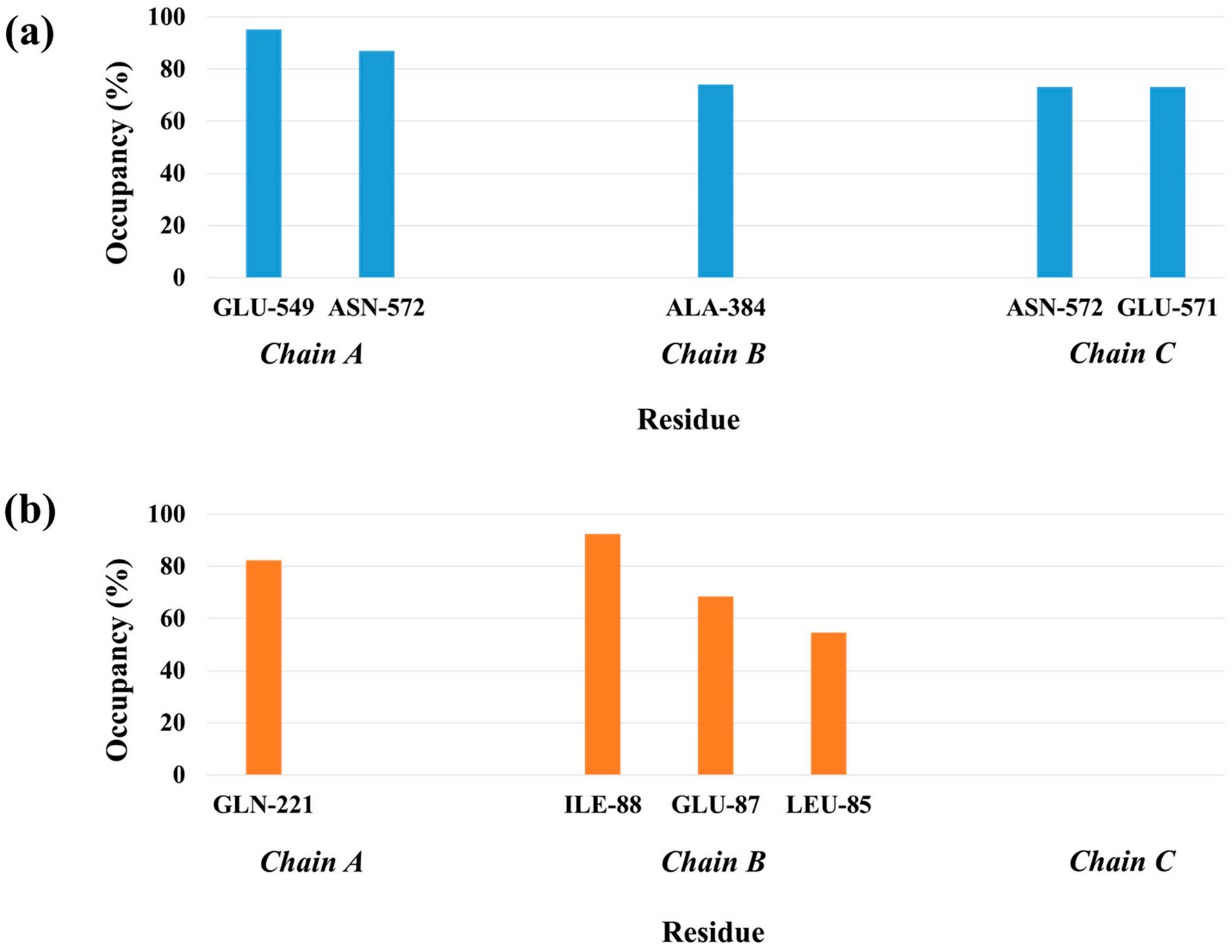

Figure S9. Hydrogen bond occupancy for residue 147 in the three-open-complex form over the entire simulation time (10 ns). The X axis represents the residues for which residue 147 exhibited hydrogen bond occupancy greater than 50% in each chain of the S protein, while the Y-axis represents the corresponding occupancy percentage. **(a)** Blue indicates the occupancy for LYS-147 in BA.2.75. **(b)** Orange indicates the occupancy for GLU-147 in BA.2.75_K147E.

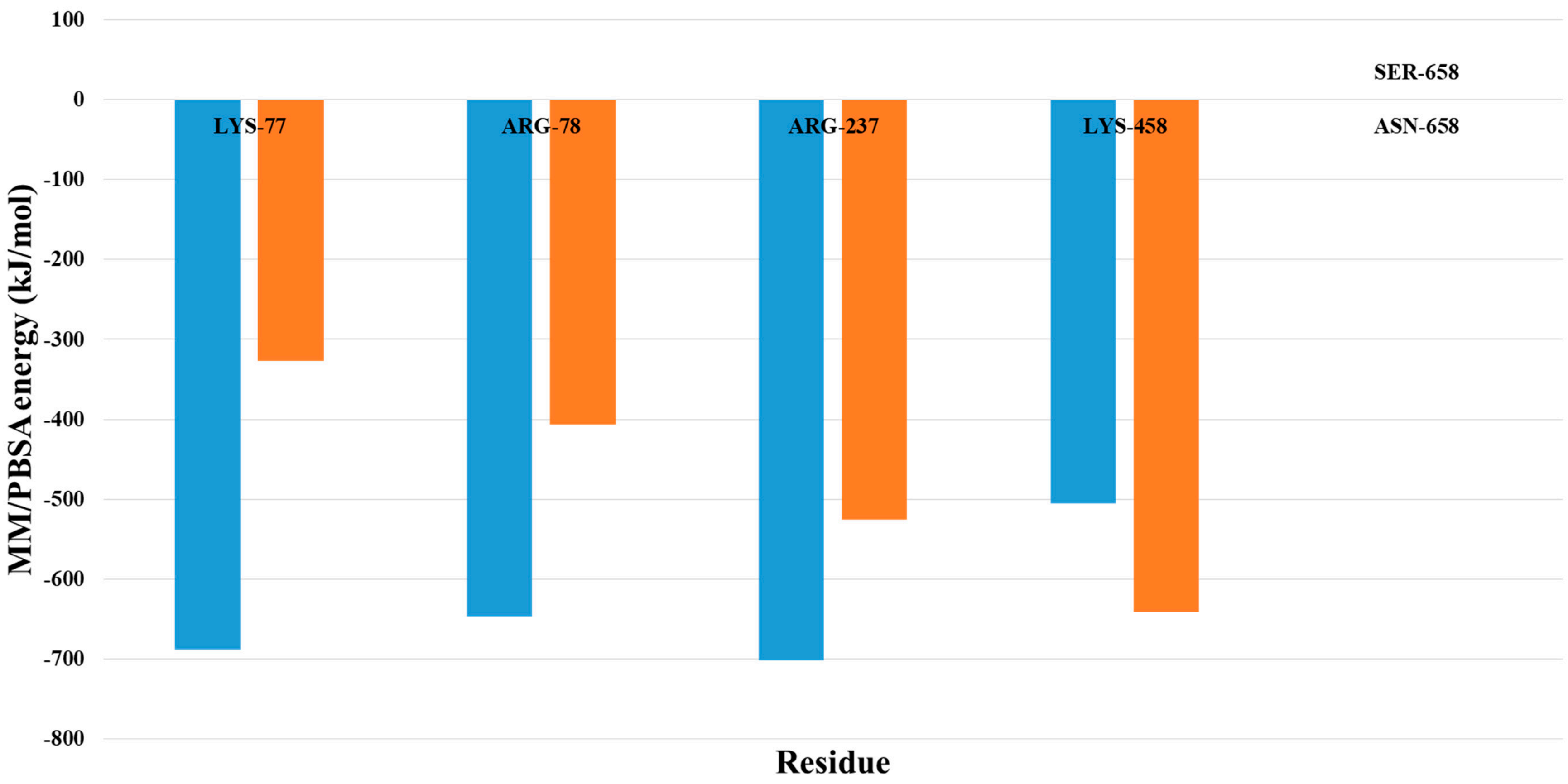

Figure S10. Contribution of residues to the binding free energy in the one-open-complex form. The X axis represents the residues with energy difference more than 100 kJ/mol, and additionally residue 658 and the Y axis represents MM/PBSA energy (kJ/mol). Blue indicates the contribution of the BA.4.6 residues to the binding free energy while orange indicates the contribution of the BA.4.6_N658S residues to the binding free energy.

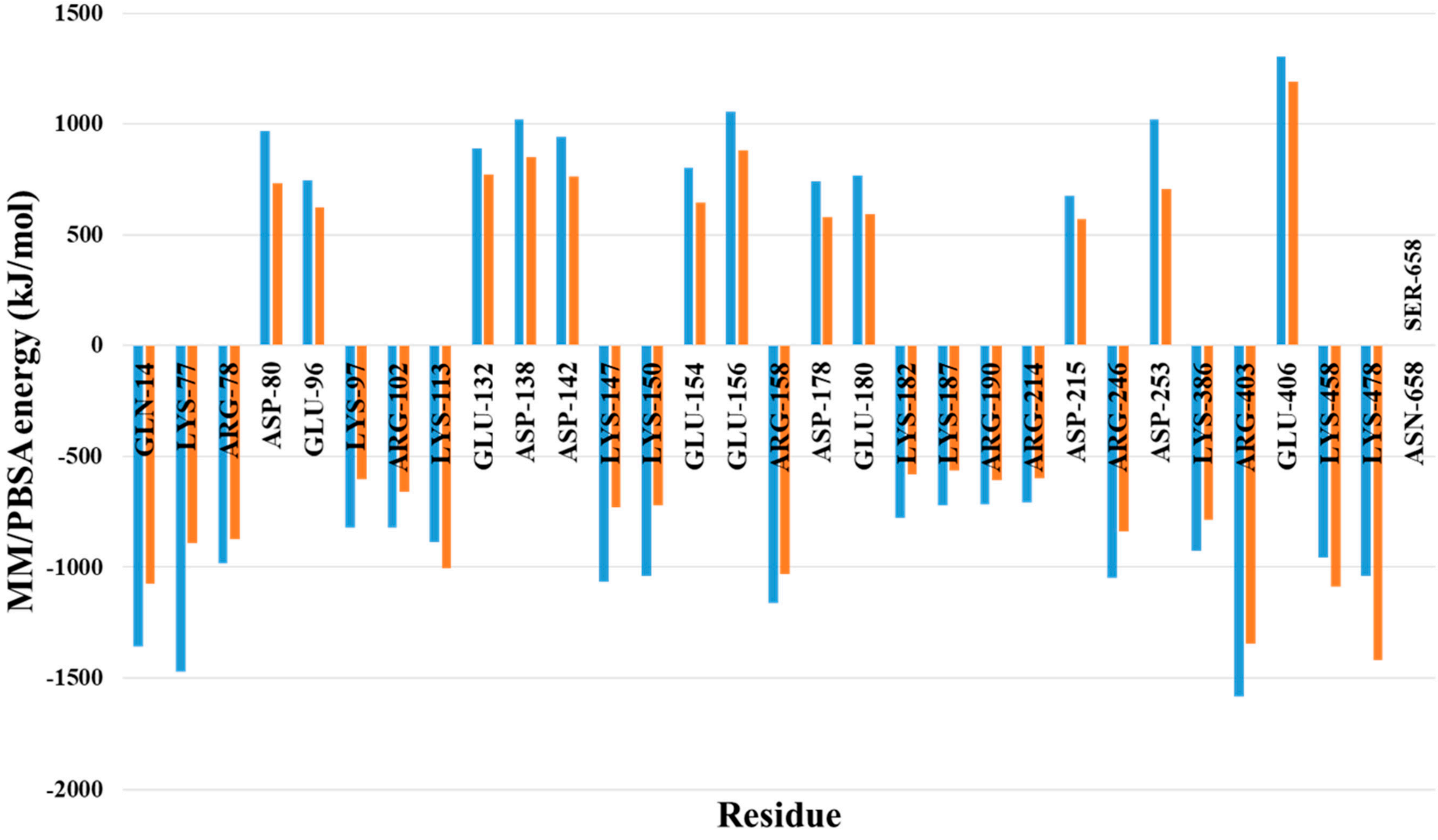

Figure S11. Contribution of residues to the binding free energy in the two-open-complex form. The X axis represents the residues with energy difference more than 100 kJ/mol, and additionally residue 658 and the Y axis represents MM/PBSA energy (kJ/mol). Blue indicates the contribution of the BA.4.6 residues to the binding free energy while orange indicates the contribution of the BA.4.6_N658S residues to the binding free energy.

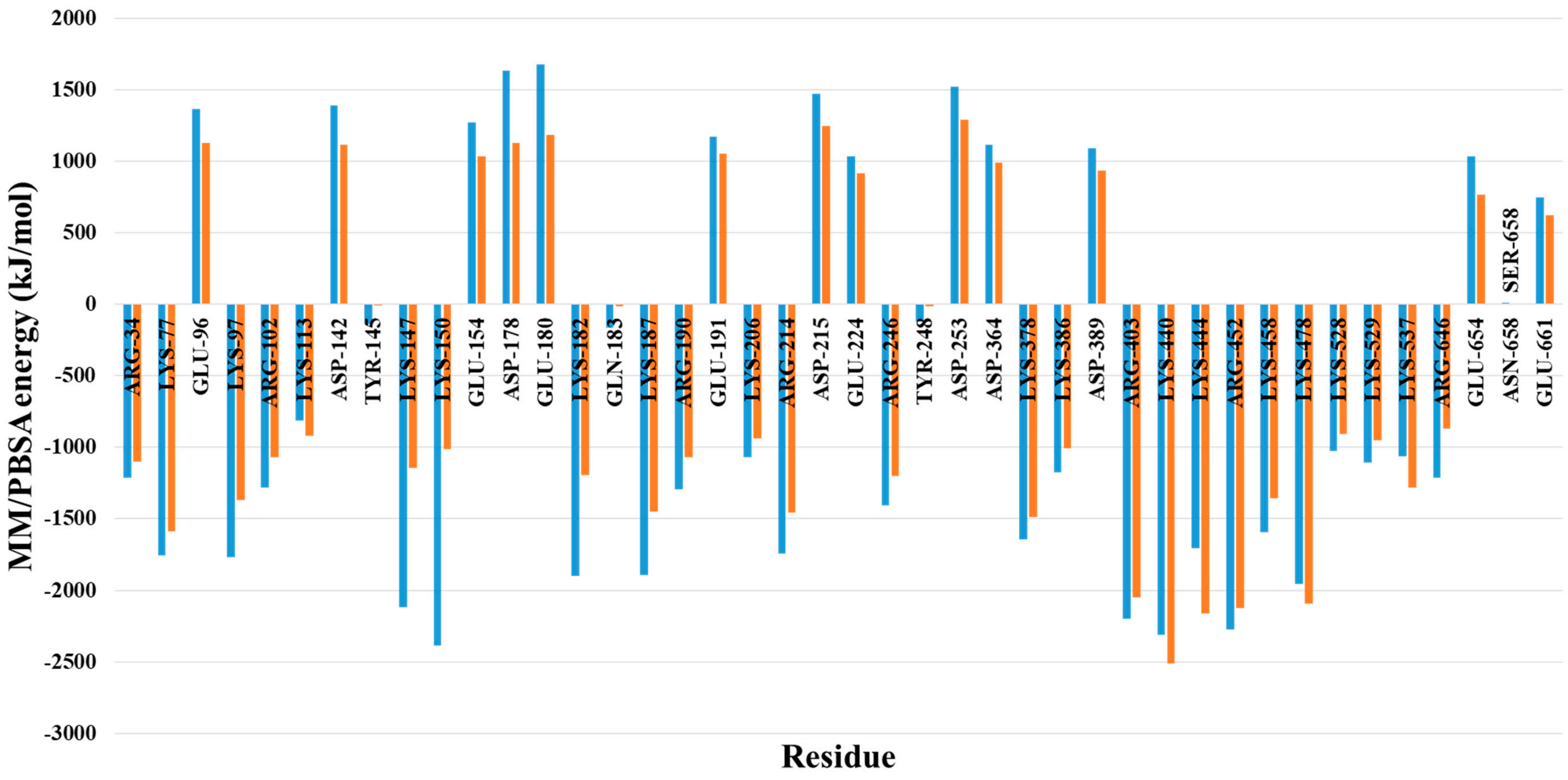

Figure S12. Contribution of residues to the binding free energy in the three-open-complex form. The X axis represents the residues with energy difference more than 100 kJ/mol, and additionally residue 658 and the Y axis represents MM/PBSA energy (kJ/mol). Blue indicates the contribution of the BA.4.6 residues to the binding free energy while orange indicates the contribution of the BA.4.6_N658S residues to the binding free energy.

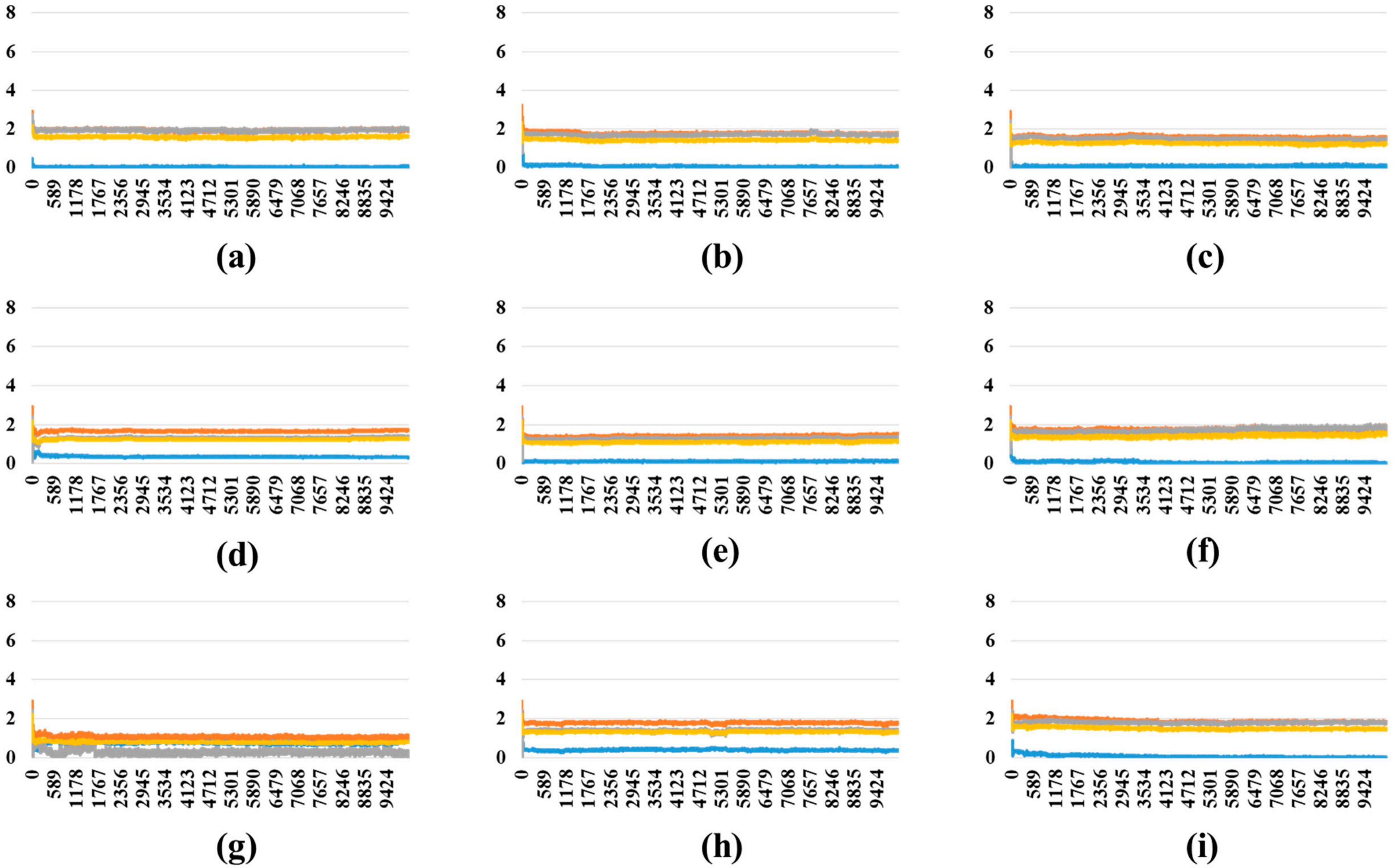

Figure S13. The standard deviations for distances between V503 residues in each chain of the S protein in the one-open-complex form during 10 ns MD simulation. **(a)** Wild type, **(b)** BA.1, **(c)** BA.2, **(d)** BA.2.12.1, **(e)** BA.4/BA.5, **(f)** BA.2.75, **(g)** BA.2.75_K147E, **(h)** BA.4.6 and **(i)** BA.4.6_N658S. X axis denotes the MD simulation time (ps) and Y axis denotes MM/PBSA energy value (kJ/mol). Blue color indicates van der Waals energy between S protein and ACE2, and orange color indicates electrostatic energy.

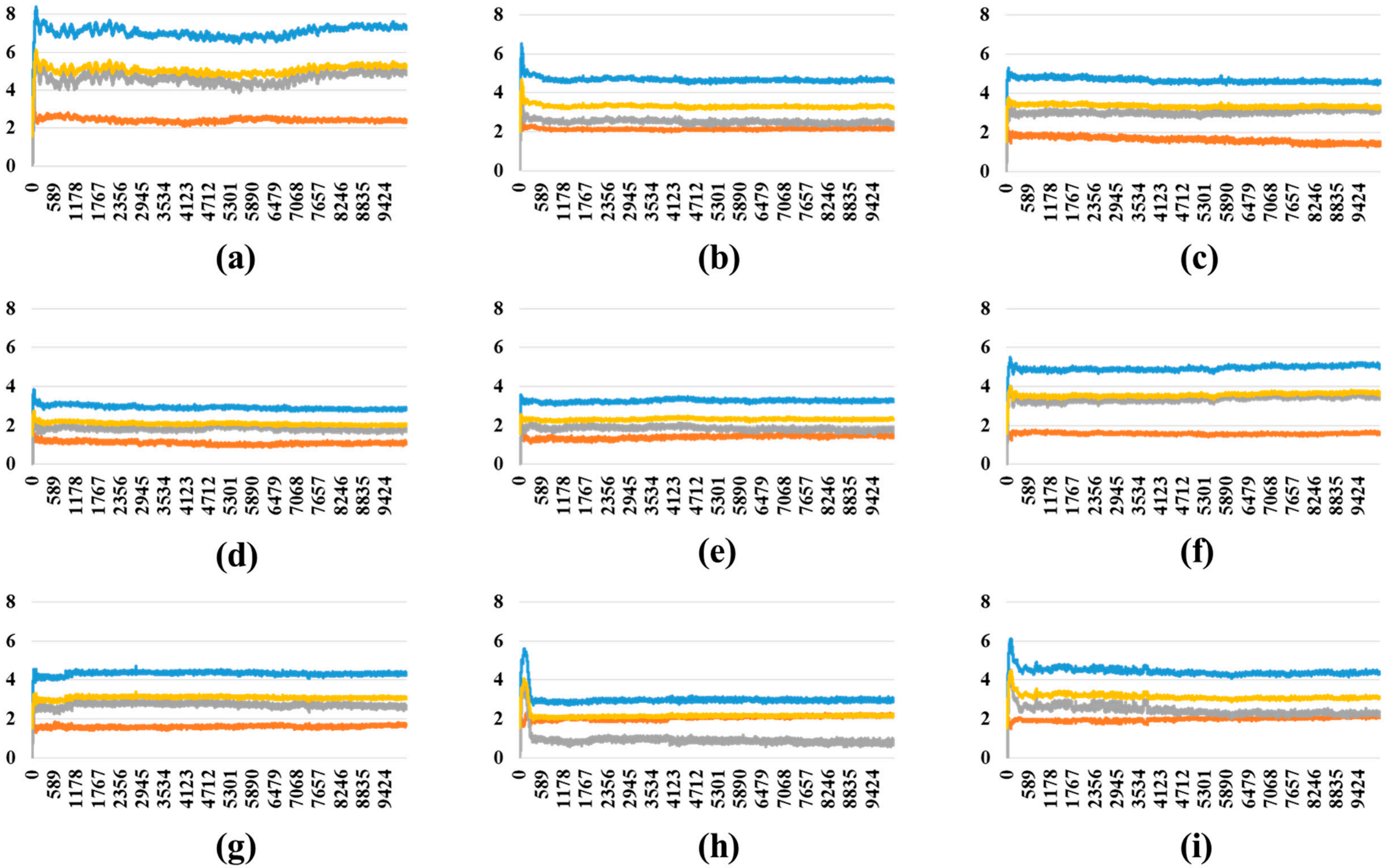

Figure S14. The standard deviations for distances between V503 residues in each chain of the S protein in the two-open-complex form during 10 ns MD simulation. **(a)** Wild type **(b)** BA.1, **(c)** BA.2, **(d)** BA.2.12.1, **(e)** BA.4/BA.5, **(f)** BA.2.75, **(g)** BA.2.75_K147E, **(h)** BA.4.6 and **(i)** BA.4.6_N658S. X axis denotes the MD simulation time (ps) and Y axis denotes MM/PBSA energy value (kJ/mol). Blue color indicates van der Waals energy between S protein and ACE2, and orange color indicates electrostatic energy.

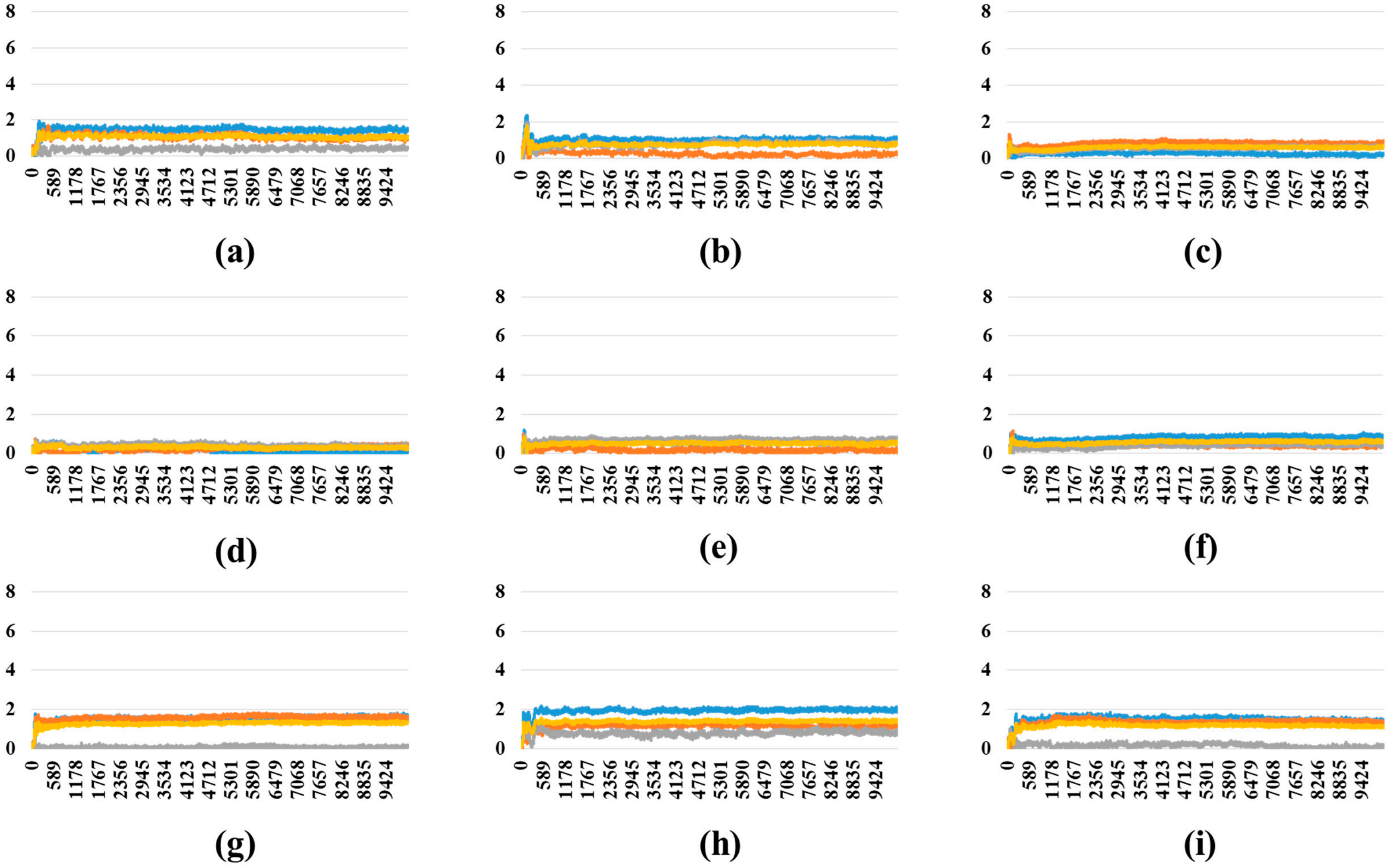

Figure S15. The standard deviations for distances between V503 residues in each chain of the S protein in the three-open-complex form during 10 ns MD simulation. **(a)** Wild type, **(b)** BA.1, **(c)** BA.2, **(d)** BA.2.12.1, **(e)** BA.4/BA.5, **(f)** BA.2.75, **(g)** BA.2.75_K147E, **(h)** BA.4.6 and **(i)** BA.4.6_N658S. X axis denotes the MD simulation time (ps) and Y axis denotes the MM/PBSA energy value (kJ/mol). Blue color indicates van der Waals energy between S protein and ACE2, and orange color indicates electrostatic energy.

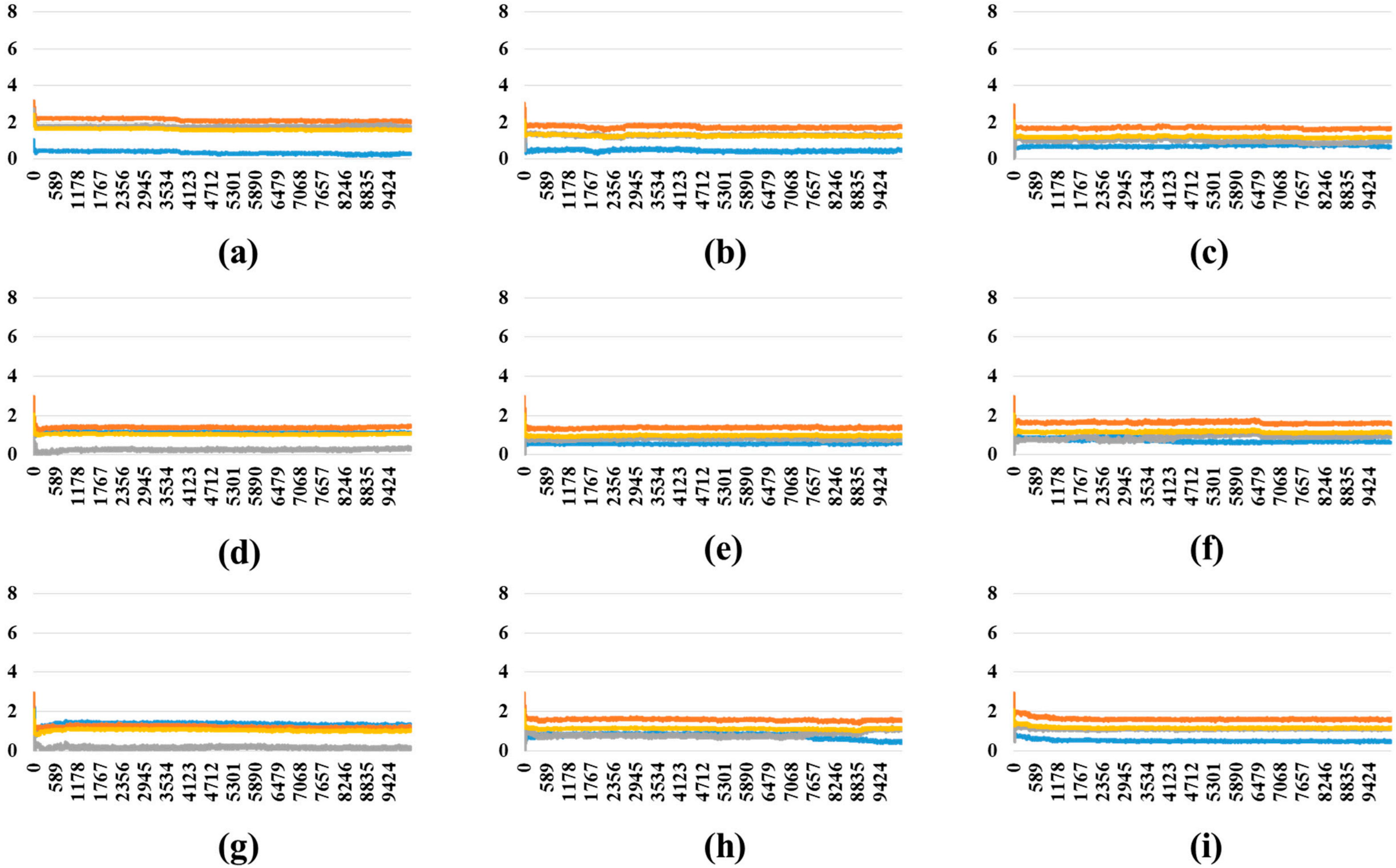

Figure S16. The standard deviations for distances between N501 residues in each chain of the S protein in the one-open-complex form during 10 ns MD simulation. **(a)** Wild type, **(b)** BA.1, **(c)** BA.2, **(d)** BA.2.12.1, **(e)** BA.4/BA.5, **(f)** BA.2.75, **(g)** BA.2.75_K147E, **(h)** BA.4.6 and **(i)** BA.4.6_N658S. X axis denotes the MD simulation time (ps) and Y axis denotes the MM/PBSA energy value (kJ/mol). Blue color indicates van der Waals energy between S protein and ACE2, and orange color indicates electrostatic energy.

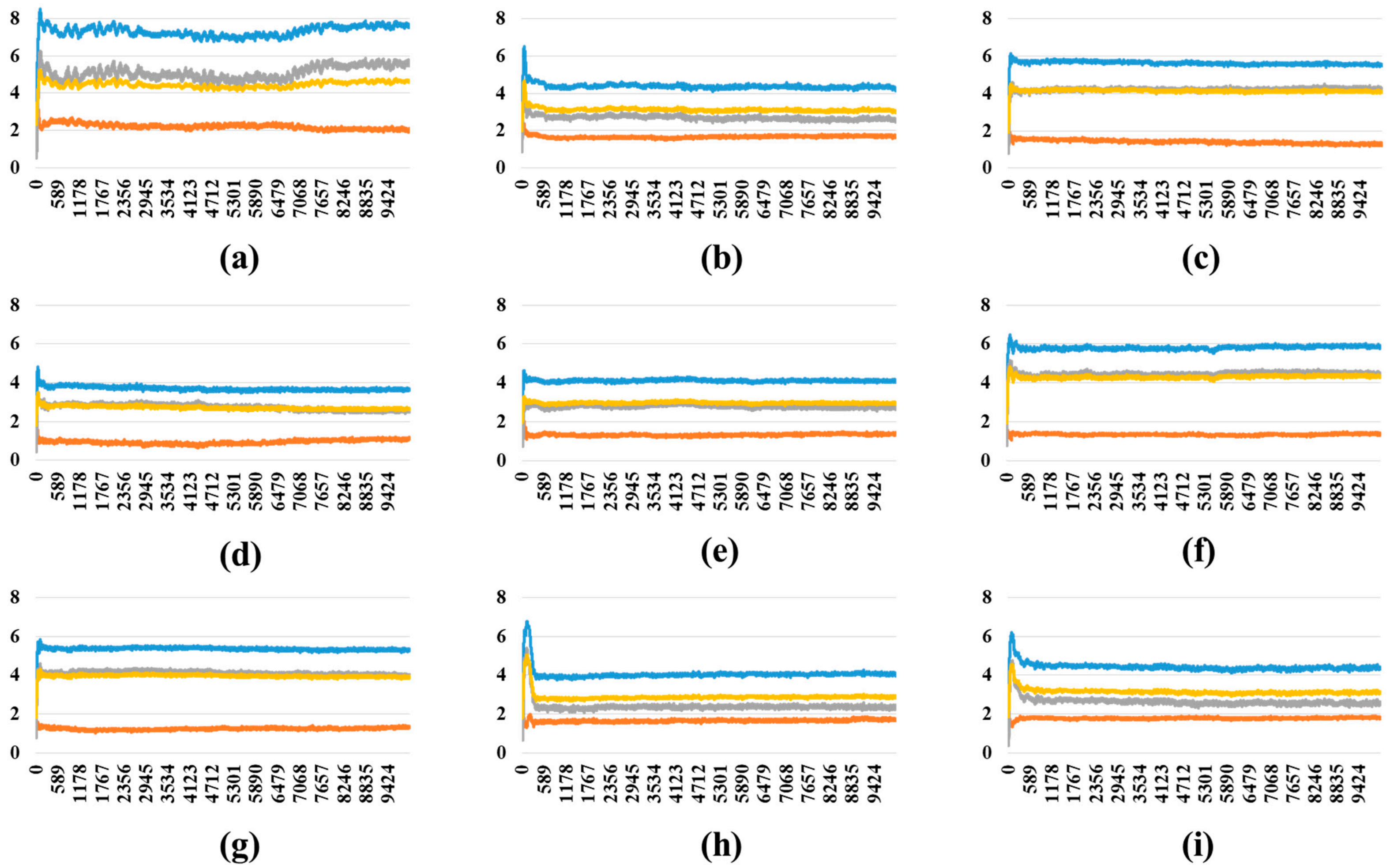

Figure S17. The standard deviations for distances between N501 residues in each chain of the S protein in the two-open-complex form during 10 ns MD simulation. **(a)** Wild type **(b)** BA.1, **(c)** BA.2, **(d)** BA.2.12.1, **(e)** BA.4/BA.5, **(f)** BA.2.75, **(g)** BA.2.75_K147E, **(h)** BA.4.6 and **(i)** BA.4.6_N658S. X axis denotes the MD simulation time (ps) and Y axis denotes the MM/PBSA energy value (kJ/mol). Blue color indicates van der Waals energy between S protein and ACE2, and orange color indicates electrostatic energy.

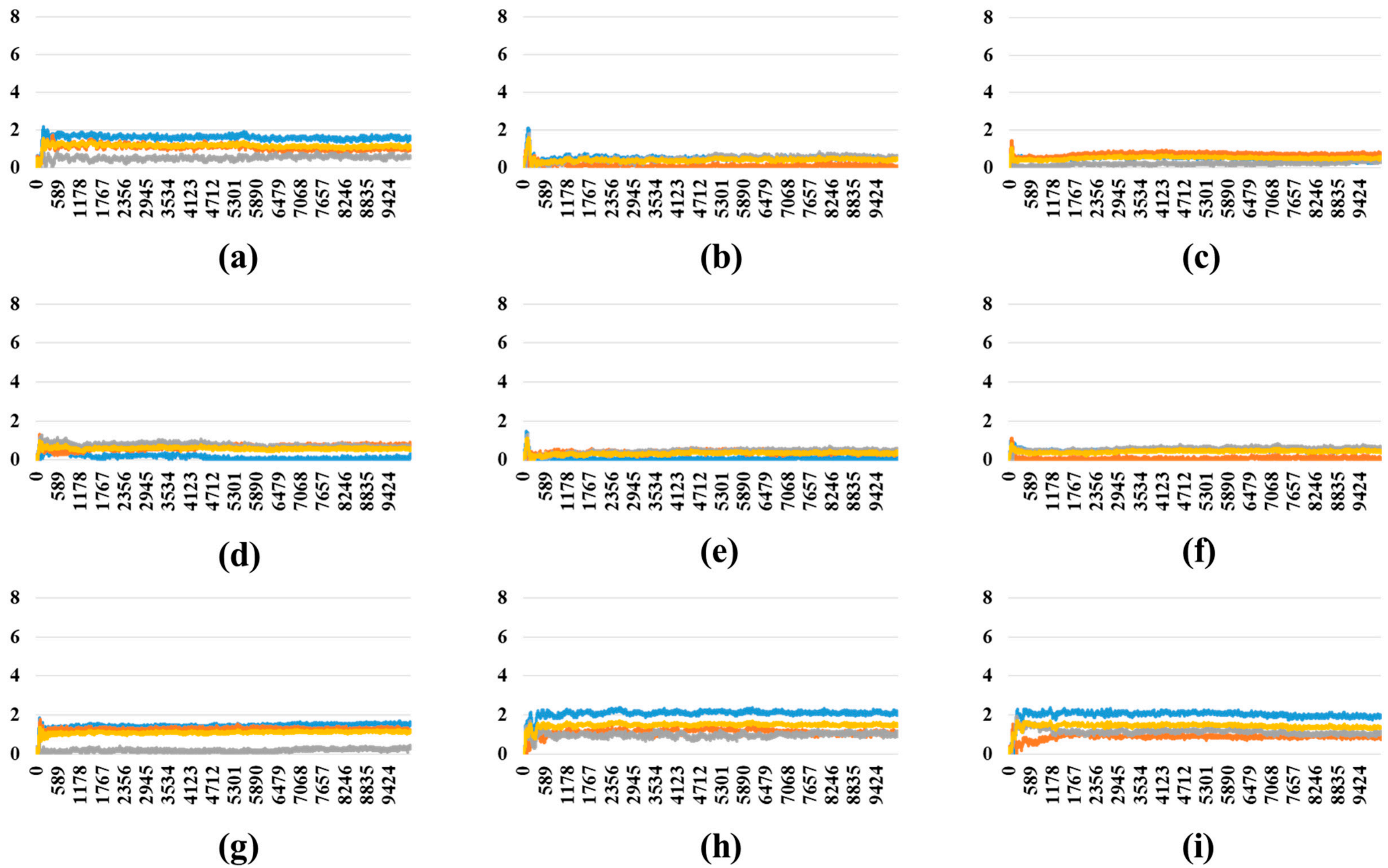

Figure S18. The standard deviations for distances between N501 residues in each chain of the S protein in the three-open-complex form during 10 ns MD simulation. **(a)** Wild type, **(b)** BA.1, **(c)** BA.2, **(d)** BA.2.12.1, **(e)** BA.4/BA.5, **(f)** BA.2.75, **(g)** BA.2.75_K147E, **(h)** BA.4.6 and **(i)** BA.4.6_N658S. X axis denotes the MD simulation time (ps) and Y axis denotes the MM/PBSA energy value (kJ/mol). Blue color indicates van der Waals energy between S protein and ACE2, and orange color indicates electrostatic energy.
